# Design of a PSO-Optimized VGG19 Convolutional Neural Network for Automatic Breast Cancer Image Classification

**DOI:** 10.3390/diagnostics16213503

**Published:** 2026-10-26

**Authors:** Precious I. Ogie, Jafar Abdollahi, Gbadegesin Adetayo Taiwo, Sunday Adeola Ajagbe, Pragasen Mudali, Hector Florez

**Affiliations:** 1 General Medicine Department, Royal Stoke University Hospital, Stoke-on-Trent ST4 6QG, UK; 2 Department of Computer Engineering, CT. C., Islamic Azad University, Tehran 14778-93855, Iran; 3 School of Computing, Science & Engineering, University of Salford, Manchester M5 4WT, UK; 4 Department of Computer Engineering, Abiola Ajimobi Technical University, Ibadan 200255, Nigeria; 5 Department and Computer Science, University of Zululand, Kwadlangezwa 3886, South Africa; 6 ITI Research Group, Universidad Distrital Francisco José de Caldas, Bogotá 110231, Colombia

**Keywords:** image classification, deep learning, breast cancer, VGG19, particle swarm optimization (PSO), histopathology

## Abstract

**Background:** Human Epidermal Growth Factor Receptor 2 (HER2) overexpression is detected in approximately 25–30% of malignant breast cancer cases and is intrinsically associated with aggressive tumor progression. Reliable assessment of HER2 status is critical for selecting optimal targeted therapies; however, conventional immunohistochemistry (HER2 SISH) evaluation predominantly relies on visual inspection, a process that is highly subjective and susceptible to inter-observer variability. To mitigate diagnostic inconsistencies, this study proposes an automated classification framework leveraging a VGG19 convolutional neural network, fundamentally enhanced via Particle Swarm Optimization (PSO) for rigorous hyperparameter tuning. The model development and evaluation in this study were performed using a purely synthetic image dataset. **Methods:** The PSO algorithm is systematically employed to optimize critical training parameters, facilitating an efficient exploration of the complex search space and significantly improving the stability of the learning process. Experimental evaluations conducted on these purely synthetic high-resolution histopathology images demonstrate that the proposed PSO-VGG19 framework substantially outperforms baseline architectures, including MobileNet. **Results:** Specifically, the optimized system achieves a classification accuracy of 98.10%, sensitivity of 98.11%, specificity of 99.37%, and an Area Under the Curve (AUC) of 99.66%. **Conclusions:** These empirical results demonstrate that integrating metaheuristic optimization with deep learning architectures yields robust computational performance for HER2 patch-level classification, serving as a foundational computational proof-of-concept for future digital pathology research.

## 1. Introduction

Breast cancer remains a pervasive and life-threatening malignancy. According to the latest Global Cancer Statistics, it accounts for 11.7% of all newly diagnosed cancer cases worldwide and is the leading cause of cancer-related mortality among women, responsible for 15.5% of such deaths [1]. The burden is particularly disproportionate in developing nations, where nearly half of the incidences occur in women under the age of 50, thereby severely impacting the working-age demographic [2,3]. Accurate prognostic evaluation and the administration of targeted therapeutic modalities are paramount for successful disease management. In this context, human epidermal growth factor receptor 2 (HER2) gene status plays a pivotal role. HER2 is a transmembrane tyrosine kinase receptor that is overexpressed in approximately 20% of breast cancer cases. This overexpression necessitates specific targeted treatments, such as trastuzumab and lapatinib, which have significantly improved patient survival rates and clinical outcomes [4,5,6,7].

Breast cancer remains one of the most pressing oncological challenges worldwide, marked by substantial global mortality rates and an urgent demand for early, precise diagnostic stratification [8]. Traditionally, Human Epidermal Growth Factor Receptor 2 (HER2) gene amplification status is determined via Silver In Situ Hybridization (SISH) or Immunohistochemistry (IHC), wherein pathologists visually evaluate cellular and punctate nuclear signals under light microscopy. According to the American Society of Clinical Oncology/College of American Pathologists (ASCO/CAP) scoring criteria, cases are categorized into four diagnostic tiers: negative (0 and 1+), equivocal (2+), and positive (3+) [9]. Despite its clinical indispensability for targeted oncological therapy, manual pathological grading suffers from inherent subjectivity, high inter-observer variability, and significant diagnostic latency [9]. Although advanced optical and hyperspectral imaging modalities have demonstrated substantial promise in characterizing ex-vivo breast tissue properties [8], the gold-standard microscopic evaluation remains highly reliant on human expertise, where misinterpretation can drastically alter treatment regimens. Consequently, deploying robust, automated computational pathology instruments to overcome human-induced variability and provide objective HER2 quantification represents a critical necessity in modern clinical workflows [9,10].

Recent advancements in Artificial Intelligence (AI) and Machine Learning (ML) have demonstrated significant potential across diverse medical domains, including X-ray imaging, infectious disease surveillance, cardiovascular diagnostics, and whole-slide histopathology [11,12,13,14,15,16,17,18,19,20]. Specifically, Convolutional Neural Networks (CNNs) have proven highly effective in autonomously learning hierarchical spatial representations from high-dimensional image data. To address the challenge of limited slide-level annotations, recent studies [21] have employed granular data extraction protocols, isolating localized discriminative pathological features from whole-slide images (WSIs) to generate robust training cohorts. Despite these advancements, a prominent limitation in prior AI-driven HER2 assessment models is the reliance on manually selected or fixed hyperparameters, which often leads to suboptimal feature extraction when handling heterogeneous HER2 SISH datasets.

The primary objective of this study is to design, optimize, and evaluate an automated deep learning framework for precise HER2 SISH scoring in breast cancer histopathology. We hypothesize that integrating Particle Swarm Optimization (PSO) with a VGG19 CNN architecture will systematically overcome the high-dimensional optimization challenges associated with hyperparameter tuning, thereby yielding superior classification performance compared to manual configuration. Rather than assigning generic benign or malignant labels, the proposed model directly predicts one of the four clinical HER2 classes (0, 1+, 2+, 3+) in strict alignment with ASCO/CAP criteria [22,23]. The core contributions of this study are threefold:

(i) The formulation of a clinically aligned classification pipeline that directly targets four-class HER2 SISH scoring to support precise treatment decisions.

(ii) The implementation of a PSO-based hyperparameter optimization scheme (tuning the learning rate, batch size, and weight initialization) for a VGG19 backbone, guided by an explicitly defined fitness function tailored for histopathological image analysis.

(iii) A rigorous systematic evaluation and comparison of the PSO-VGG19 framework against a widely adopted lightweight baseline model (MobileNet) and previous non-optimized state-of-the-art methods, establishing a robust proof-of-concept for evolutionary hyperparameter tuning in histopathological image analysis.

To contextualize the proposed methodology within the current scientific landscape, Table 1 presents a comprehensive comparative analysis of existing histopathology-based detection methods and their empirical performance metrics. Furthermore, Table 2 provides a structured summary of other representative AI-driven diagnostic frameworks applied to breast cancer histopathology and other complex medical imaging domains, highlighting the gap that our PSO-optimized architecture aims to bridge [24,25,26,27,28,29,30,31].

To address the inherent challenge of relying on slide-level labels, which often lack spatial precision, a more granular data extraction protocol was adopted. This protocol, originally introduced for the scarce HER2 dataset [31], guided the selection of localized training data. Specifically, representative regions were meticulously extracted from 53 SISH-stained whole-slide images (WSIs) at a low resolution (level 6) [31]. The primary objective of this procedure was to isolate the most discriminative pathological features corresponding to each HER2 category. This spatial localization was achieved by mapping the exact coordinates of the target regions on the SISH-stained slides, utilizing structural reference data from corresponding Hematoxylin and Eosin (H&E) stained slides, as detailed in [31].

The integration of machine learning and deep learning has profoundly advanced diverse domains of healthcare, ranging from clinical prognosis to complex medical image analysis. In clinical and toxicological settings, predictive machine learning models have been successfully employed to forecast patient outcomes following bupropion [32] and diphenhydramine [33] exposures, as well as for the early detection of neurodevelopmental conditions such as autism spectrum disorder [23]. Transitioning to computer-aided diagnosis in medical imaging, specialized neural architectures have demonstrated remarkable capabilities across various modalities. Notable examples include the application of LeNet for lung cancer classification on CT scans [34], the utilization of VGG16 integrated with NestNet for identifying brain metastasis in MRIs [30], GBO-optimized CNNs for infectious disease detection like COVID-19 [29], and the deployment of multi-modal Swin Transformers leveraging both spatial and visual features for complex wound classification [22]. More specifically within breast cancer research, generative adversarial networks (cGANs) have been utilized to synthesize breast MRI data for radiogenomic mutation predictions [31], while novel hybrid multi-scale adaptive convolutional transformers (MACT-CSA) with self-supervised learning have been proposed to successfully mitigate domain shifts and annotation scarcity in histopathological breast cancer classification [21]. Building upon these versatile applications of artificial intelligence in medical diagnostics, our current study specifically focuses on the enhancement of HER2 status classification.

In addition to the four standard HER2 diagnostic classes (scores ranging from 0 to 3+), a discrete background class was incorporated into the dataset. This class encompasses textured tissue regions completely devoid of specific HER2 in situ hybridization markers (i.e., lacking any distinct black or dark grey punctate silver signals). Ultimately, in alignment with the benchmark data curation established by the HER2 Challenge Contest [34], this rigorous selection process yielded a total of 119 annotated regions, with dimensions varying significantly from 22,184 to 59,006,976 pixels.

Beyond breast cancer histopathology, the efficacy of advanced deep learning and metaheuristic-optimized frameworks has been extensively validated across a broad spectrum of complex medical diagnostic tasks. Recent studies have demonstrated the robustness of ensemble machine learning for cervical cancer diagnosis [34], while hybrid optimization models, such as Harris Hawks Optimization combined with VAE-Transformers, have significantly enhanced Electronic Health Record (EHR) auditing for diabetes management [35]. In pulmonary oncology, convolutional architectures including VGG networks and Xception have been successfully employed to accurately classify lung cancer nodules from CT scans [36,37,38]. Furthermore, deep learning techniques have proven critical in the timely prediction of pandemic-prone infectious diseases [39], super-resolution ultrasound imaging of microvessels [40], and complex MRI segmentation for sports-related knee joint injuries using ant-colony optimization [41]. Advanced image processing has also extended to surgical instrument segmentation in minimally invasive environments [42] and dynamic time-warping optimizations for complex clinical time-series [43,44]. Even within breast cancer analytics, parallel progress is evident in alternative imaging modalities, such as dual-branch Faster R-CNNs for breast ultrasound detection [45,46,47,48,49,50]. Collectively, these cross-domain successes highlight the critical role of architecture optimization—a principle that motivated our choice of a PSO-optimized VGG19 network for HER2 status classification.

**Table 2:** Summary of representative histopathology-based breast cancer detection methods.

Study	Methodology	Dataset	Performance Metrics	Limitations
Araujo et al. [24]	CNN-based histology classification	BACH	Acc: 77.8%, SE: 87.4%, SP: 95.6%	Limited depth; no optimization
Pan et al. [25]	Deep CNN	BreakHis	Acc: 92.2%, SE: 90%, SP: 87%	No hyperparameter tuning
Janowczyk et al. [26]	Deep hierarchical learning	Private	Acc: 82.2%	Small dataset
Sudharshan et al. [27]	Multiple instance learning	BreakHis	Acc: 92.3%	Patch-level only
Yang et al. [28]	PSO-optimized hybrid features	BHI	Acc > 94%	Not HER2 specific
Kiziloluk and Sert [29]	GBO-optimized CNN, COVID CCD Net	COVID-19 & Epistroma	Acc > 97%	Not applied to HER2
Alshammari et al. [30]	VGG16 with NestNet for brain metastasis classification	Brain MRI	Acc: 93.74%, SE: 92.10%	Not HER2-related; specific to brain metastasis detection
Huang [31]	cGAN for synthetic MRI generation to predict gene mutation status (Radiogenomics)	Breast MRI (TCIA/TCGA)	AUC up to 0.95 for predicting TP53, PIK3CA, CDH1 mutations	Focus on radiogenomic data generation, not direct HER2 classification
Qaiser et al. [49]	Automated HER2 scoring benchmarking (Deep learning & ensemble models)	HER2 Challenge Contest (86 WSIs/119 annotated regions)	Automated scoring agreement comparable to / exceeding pathologist consensus	Focused on IHC modalities; susceptible to inter-observer variability in manual ground truth

In this study, a unified deep learning framework was designed to execute the four-class HER2 SISH scoring directly, thereby eliminating the necessity for disparate models for individual HER2 categories and significantly streamlining the clinical decision-making pipeline. While deep learning models are widely recognized for their efficacy in medical image analysis, this framework is specifically tailored for the automated classification of HER2 nuclear punctate signal patterns in breast cancer tissue architectures. Our primary objective is to evaluate the diagnostic efficacy of two robust architectural approaches: a highly optimized baseline and an advanced hybrid metaheuristic model. By employing a single comprehensive diagnostic tool, the proposed approach minimizes computational redundancy and reduces overall decision-making latency.

The proposed model architecture leverages the VGG19 convolutional neural network optimized via Particle Swarm Optimization (PSO). The standard VGG19 architecture comprises 19 learnable layers (16 convolutional layers and 3 fully connected layers) and is structurally designed to process high-dimensional visual data. The network utilizes uniform 3×3 convolutional filters to systematically extract hierarchical morphological features from 224×224 pixel input images. These localized feature maps are subsequently aggregated by the fully connected layers to map the extracted attributes to their respective predictive classes. While VGG19 inherently possesses robust feature-extraction capabilities due to its depth, its performance is highly sensitive to hyperparameter configurations. By integrating PSO, the hybrid model autonomously fine-tunes critical hyperparameters—specifically the learning rate, batch size, and network momentum. This optimization leverages the global search capabilities of the PSO algorithm, allowing the VGG19 model to avoid local minima and reliably differentiate between nuanced signal clustering (e.g., distinguishing between 1+ and 2+ cases).

Although auxiliary experiments were initially considered, this study systematically focuses on comparing the PSO-optimized VGG19 classifier against a standard MobileNet architecture to benchmark performance. The operational pipeline of the model consists of three primary stages:

- **Data Pre-Processing:** Preparation and standardization of the raw HER2 SISH input images.
- **Feature Extraction:** Utilization of the deep convolutional layers to isolate diagnostically relevant nuclear signal patterns.
- **Classification:** Assignment of the extracted feature vectors to one of the four predefined ASCO/CAP HER2 categories.

**Figure 1:**
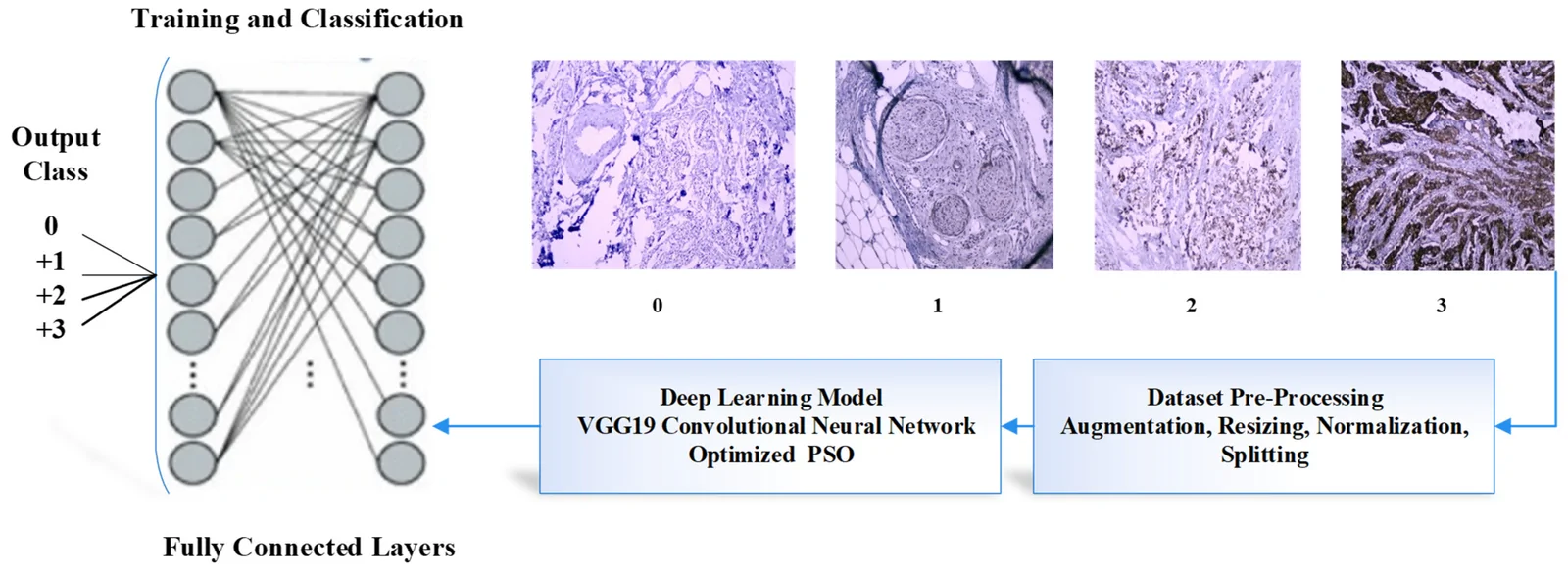
Block diagram of the proposed multi-classification Multi-classification Breast Cancer Model.

A schematic block diagram of the proposed multi-classification system is illustrated in Figure 1.

### VGG19 + CNN Deep Model

The data preprocessing stage incorporates several rigorous operations to ensure input consistency and model generalization. Initially, all WSI-derived patches are resized to a standard spatial dimension of 224×224 pixels. The dataset is subsequently partitioned stochastically into a 70:30 ratio, designating 70% of the images for the training phase and 30% for independent validation. This stochastic partitioning ensures visual and morphological diversity across both subsets. Following the conversion of the images into standardized pixel arrays, data normalization is performed by rescaling pixel intensity values to a continuous range of [0,1]. This normalization accelerates gradient convergence and standardizes the input distribution.

To further fortify the network against overfitting and enhance classification reliability, a dual-augmentation strategy is employed. Spatial transformations—including horizontal/vertical flipping, controlled angular rotations, and subtle geometric skewing—are applied both during the initial dataset aggregation (incorporating CGAN-augmented samples) and dynamically during the preprocessing phase. This exponential expansion of the training distribution ensures that the model is robust to the orientation and translational invariances inherently found in microscopic tissue samples. Ultimately, the integration of VGG19’s profound feature representation with PSO’s precision tuning produces a robust pipeline capable of achieving high-accuracy detection and accurate subtyping of breast cancer markers.

**A. Optimization Using Particle Swarm Optimization (PSO):**

In this study, the Particle Swarm Optimization (PSO) algorithm is employed to systematically fine-tune the hyperparameters of the VGG19 architecture. The primary objective of the integrated PSO-VGG19 framework is to minimize the classification loss over the histopathology training dataset, thereby enhancing the model’s discriminative capacity for HER2 scoring. Let f(⋅) denote the VGG19 network parameterized by the candidate hyperparameter vector $X_{i}$. The objective function $J\left(X_{i}\right)$, based on the categorical cross-entropy loss, is mathematically formulated as:

(1) $$J\left(X_{i}\right)=-\frac{1}{N}\sum_{j=1}^{N}\sum_{k=1}^{C}y_{j,k}log\left({\hat{y}}_{j,k}\left(X_{i}\right)\right)$$

where N is the total number of training samples, C=4 represents the number of target clinical classes (HER2 0, 1+, 2+, 3+), $y_{j,k}$ is the ground truth binary indicator for class k, and ${\hat{y}}_{j,k}\left(X_{i}\right)$ is the predicted probability generated by the model. Consequently, the global optimal hyperparameters are dynamically derived by the swarm as $G_{best}=\arg{min}_{X}J\left(X\right)$.

**B. Objective Function and VGG19 Integration**

**Figure 2:**
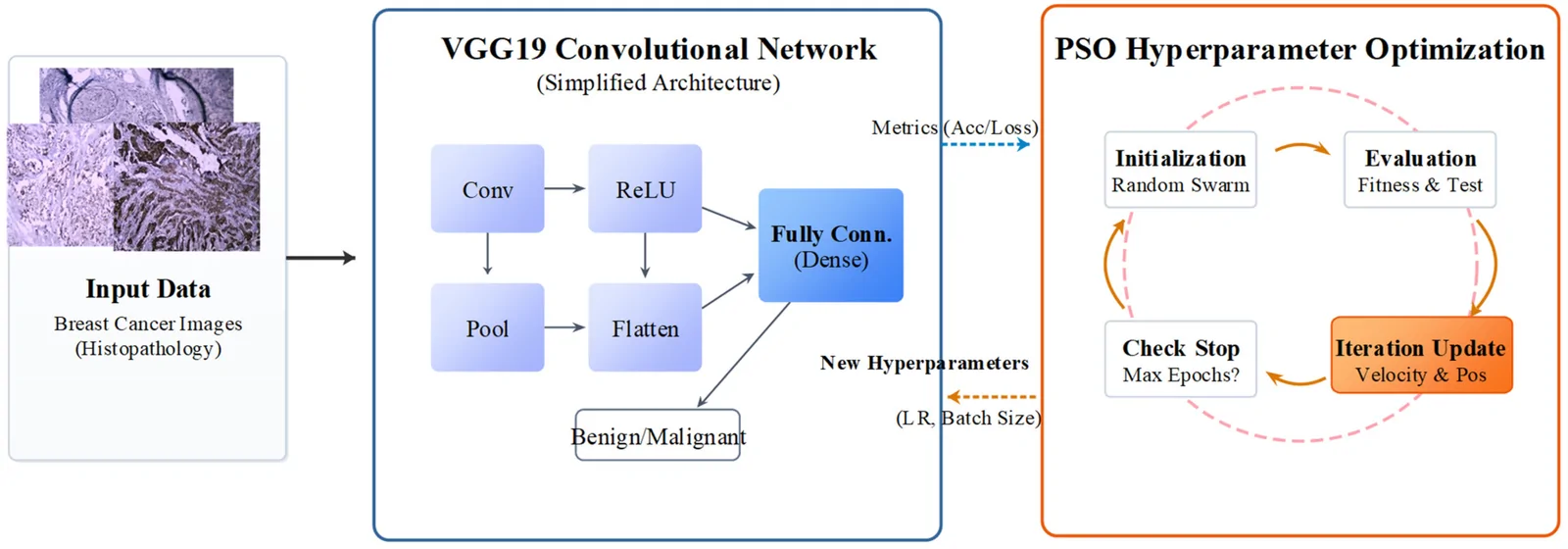
Schematic of the PSO Hyperparameter Optimization Loop Integrated with the VGG19 Convolutional Network.

The primary objective of the integrated PSO-VGG19 framework is to minimize the classification loss over the histopathology training dataset. Let f(⋅) denote the VGG19 network parameterized by the optimized vector $x_{i}$. The objective function $J\left(x_{i}\right)$, based on the categorical cross-entropy loss, is formulated as:

(2) $$J\left(x_{i}\right)=-\frac{1}{N}\sum_{j=1}^{N}\sum_{k=1}^{C}y_{j,k}log\left({\hat{y}}_{j,k}\left(x_{i}\right)\right)$$

where N is the total number of training samples, C represents the number of target classes (e.g., HER2 status categories), $y_{j,k}$ is the ground truth binary indicator (0 or 1), and ${\hat{y}}_{j,k}\left(x_{i}\right)$ is the predicted probability generated by the model. Consequently, the global optimal hyperparameters are dynamically derived as $g_{best}=\arg{min}_{x}J\left(x\right)$. Figure 2 presents the schematic of the PSO Hyperparameter Optimization Loop Integrated with the VGG19 Convolutional Network.

In this study, a modified VGG19 architecture was employed for the feature extraction and classification of HER2 histopathological images into four distinct classes (0, 1+, 2+, and 3+). The convolutional layers extract spatial hierarchies of features using the following operation:

(3) $$y_{i,j}=\sum_{m}\sum_{n}w_{m,n}\cdot x_{i+m,j+n}+b$$

To introduce non-linearity, the Rectified Linear Unit (ReLU) activation function is applied to the feature maps:

(4) f(x)=max(0,x)

Dimensionality reduction is achieved through Max-pooling layers, which retain the most salient features:

(5) $$y_{i,j}=\underset{m,n}{max}\left(x_{i+m,j+n}\right)$$

Finally, the dense layers map the extracted features to the target classes. For the terminal classification layer, a SoftMax activation function is utilized to compute the probability distribution across the four HER2 classes, defined as:

(6) $$P\left(y=k|x\right)=\frac{e^{z_{k}}}{\sum_{j=1}^{4}e^{z_{j}}}$$

where k∈{1,2,3,4} corresponds to the four HER2 scoring categories, and z represents the logits from the preceding dense layer.

**C. Model Training and Validation**

The PSO algorithm was configured with a swarm size of 20 particles and executed for a maximum of 30 iterations. The search space was strictly two-dimensional, tasked specifically with optimizing the learning rate and batch size, with mean validation accuracy serving as the fitness function. Based on preliminary empirical benchmarking, the Adamax (fixed) optimizer was selected *a priori* as the fixed optimization algorithm, allowing the PSO to dedicate its entire computational budget to the aforementioned hyperparameters. The definitive hyperparameters yielded by the PSO—which were subsequently applied to generate all reported comparative results—included the Adamax (fixed) optimizer, a learning rate of 0.00006, and a batch size of 64. To formulate the clinical task as a four-class classification problem (HER2 0, 1+, 2+, and 3+), the classification head of the proposed VGG19 architecture was specifically tailored. The final fully connected layer was configured with four output neurons corresponding to the mutually exclusive HER2 categories. A **softmax** activation function ($\sigma {\left(z\right)}_{i}=\frac{e^{z_{i}}}{\sum_{j=1}^{4}e^{z_{j}}}$) was applied to this terminal layer to compute the standard probability distribution across all four classes. Consistently, the network weights were optimized by minimizing the **categorical cross-entropy** loss function ($L=-\sum_{i=1}^{4}y_{i}log\left({\hat{y}}_{i}\right)$), ensuring methodological rigor for this multi-class pathological evaluation. Models were developed using two deep learning architectures: MobileNet and VGG19. Each architecture was connected to a final fully connected layer configured with four neurons, corresponding to the four distinct HER2 categories (0, 1+, 2+, and 3+). A **softmax** activation function ($\sigma {\left(z\right)}_{i}=\frac{e^{z_{i}}}{\sum_{j=1}^{4}e^{z_{j}}}$) was applied to this terminal layer to output a normalized class probability distribution. Consequently, **categorical cross-entropy** ($L=-\sum_{i=1}^{4}y_{i}log\left({\hat{y}}_{i}\right)$) was strictly employed as the loss function throughout the learning process to optimize the multi-class classification performance.

Models were developed using two deep learning architectures: MobileNet (acting as the baseline) and VGG19 (the proposed architecture). To explicitly address the four-class HER2 HER2 SISH scoring problem (classes 0, 1+, 2+, and 3+), both architectures were terminated with a fully connected classification head employing a **SoftMax** activation function comprising exactly four neurons. Consequently, **categorical cross-entropy** was utilized as the loss function throughout the learning process. The experimental protocol and hyperparameters were strictly defined and optimized using Particle Swarm Optimization (PSO) for the VGG19 model. The PSO algorithm was configured with a swarm size of 20 particles and executed for 50 iterations, specifically tasked with optimizing the learning rate, optimizer selection, and batch size to minimize the validation loss as the stopping criterion. The definitive hyperparameters yielded by the PSO—which were subsequently applied to generate all reported comparative results—included the **Adamax** (fixed) optimizer, a learning rate of **0.00006**, and a batch size of **64**.

Training for both the PSO-optimized VGG19 and the baseline MobileNet was conducted for exactly **50 epochs**, as this duration demonstrated optimal convergence and stabilization of both loss and accuracy metrics without inducing overfitting. To enhance the robustness of the dataset prior to CGAN augmentation, standard spatial transformations were applied to the training set, including a 10% random shift, a 0.1-radian random rotation, and a 0.1-factor random brightness adjustment. All models were initialized with fixed random seeds to ensure total reproducibility of the experimental protocol.

To rigorously prevent any potential data leakage, the purely synthetic dataset was strictly partitioned at the image level prior to any data augmentation procedures. The dataset was split into a training set (60% of images), a validation set (20% of images), and a testing set (20% of images). The Conditional Generative Adversarial Network (CGAN) was exclusively trained using only the designated training set to synthesize minority class images. Consequently, the validation and test sets remained completely unseen by both the CGAN during augmentation and the subsequent PSO-VGG19 model during training, preserving the integrity of the evaluation phase. The proposed PSO-VGG19 network was compiled using the Categorical Cross-Entropy loss function to accommodate the multi-class nature of the HER2 scoring system. The network weights were optimized using the Adamax optimizer, initialized with a learning rate of 0.00006. The training process was executed with a batch size of 64 over exactly 50 epochs, which, as demonstrated by the convergence graphs, provided an optimal balance between loss minimization and prevention of overfitting.

**Figure 3:**
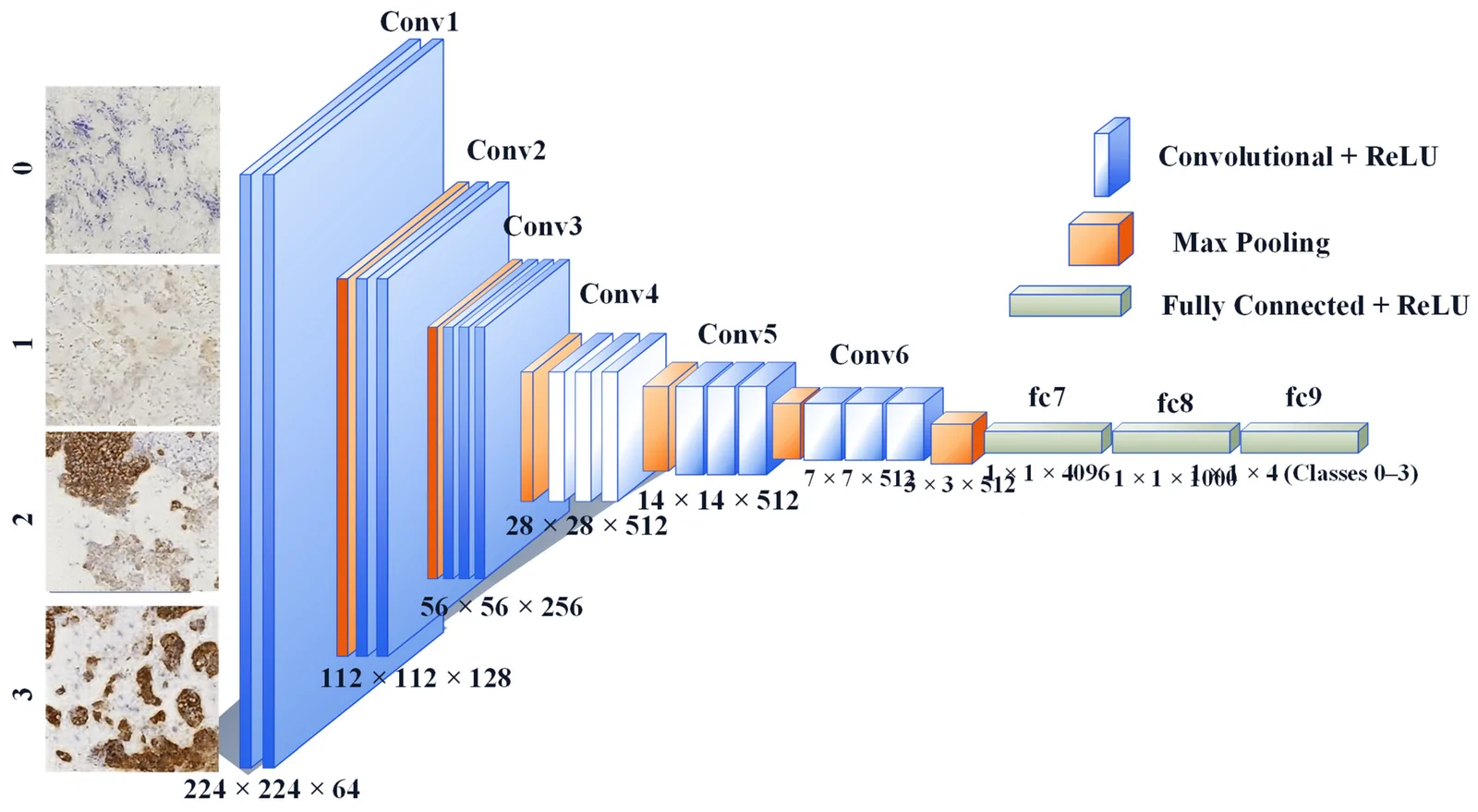
Detailed Architecture of the Proposed VGG19 + CNN Model for Histopathological Feature Extraction.

The primary objective of this phase was to accurately classify HER2 expression levels utilizing the designated histopathological datasets. To ensure robust evaluation and mitigate overfitting, the data was partitioned using stratified five-fold cross-validation, upon which the models were developed and rigorously fine-tuned. To artificially expand the training cohort and enhance model generalizability, on-the-fly data augmentation techniques were applied, including a 10% random spatial shift, random rotations up to 0.1 radians, and a random brightness adjustment factor of ±0.1 (or 10%). During the internal validation phase, a model checkpointing strategy was implemented to continuously monitor the validation loss, ultimately retaining the optimal model weights corresponding to the minimum loss function value across the 50 epochs. Furthermore, a tiered evaluation framework was established to systematically assess the clinical applicability of the proposed architectures. As delineated in Table 3, this framework encompasses sequential stages ranging from initial training on constrained datasets to prospective full-scale clinical deployment. Figure 3 is the detailed Architecture of the Proposed VGG19 + CNN Model for Histopatho-logical Feature Extraction.

**D. VGG19 Architecture and Mathematical Foundations**

To achieve integration into clinical diagnostics, a classifier must successfully navigate the tiered evaluation framework outlined in Table 3. Our proposed architecture leverages the VGG19 network, a profound Convolutional Neural Network (CNN) comprising 19 learnable layers. Pre-trained on the expansive ImageNet dataset [49]—encompassing over one million images across 1000 distinct object categories VGG19 provides a highly optimized foundation for robust feature extraction.

The architecture incorporates 16 convolutional layers, interspersed with max-pooling layers, and culminates in a customized fully connected classifier head. Mathematically, the discrete 2D convolution operation for an input matrix X and a learnable filter W of spatial dimensions M×N (where M=3 and N=3 throughout the VGG19 convolutional blocks) is defined as:

(7) $$\left(X*W\right)\left(i,j\right)=\sum_{m=0}^{M-1}\sum_{n=0}^{N-1}X\left(i,-mj-n\right)W\left(m,n\right)$$

Subsequent to each convolutional operation, the Rectified Linear Unit (ReLU) activation function is applied. This introduces critical non-linearity into the network, facilitating the modeling of highly complex diagnostic patterns. The ReLU function is formally expressed as:

(8) f(x)=ax(0,x)

To systematically downsample the spatial dimensions of the intermediate feature maps—thereby mitigating computational complexity and inducing spatial translation invariance—the architecture employs max-pooling layers. Utilizing a 2×2 pooling window with a stride of s=2, the max-pooling operation is mathematically formulated as:

(9) $$Y\left(i,j\right)=\underset{m,n\in \left\{0,1\right\}}{max}X\left(i,\cdot s+mj\cdot s+n\right)$$

To specifically resolve the four-class HER2 scoring task, the terminal fully connected layer of our modified VGG19 architecture is engineered with exactly four neurons, followed by a Softmax activation function. This function transforms the raw output logits ($Z_{k}$) into a normalized probability distribution across the four distinct target classes (0, 1+, 2+, 3+), defined as:

(10) $$P\left(y=k|Z\right)=\frac{e^{Z_{k}}}{\sum_{j=1}^{4}e^{Z_{j}}}\;\mathrm{for}\;k\in \left\{1,2,3,4\right\}$$

Ultimately, the network’s learnable parameters were iteratively optimized utilizing the Categorical Cross-Entropy loss function, perfectly aligning the output topology with the multi-class clinical diagnostic objective. To ensure mathematical clarity, a comprehensive nomenclature table detailing all key symbols is presented in Table 4.

Following the extraction of high-dimensional spatial features, the data propagates through the fully connected (dense) layers of the VGG19 architecture. These layers integrate the hierarchical features to facilitate the final diagnostic decision. To align with our specific multi-class clinical objective, the terminal layer employs a Softmax activation function, which maps the network’s raw logits into a normalized probability distribution across the four distinct HER2 expression classes (0, 1+, 2+, and 3+):

(11) $$P\left(y=k|Z\right)=\frac{e^{Z_{k}}}{\sum_{j=1}^{4}e^{Z_{j}}}$$

During the backpropagation phase, the network’s learnable parameters (weights W and biases b) are iteratively updated to minimize the Categorical Cross-Entropy loss function:

(12) $$L=-\sum_{c=1}^{M}y_{o,c}log\left(p_{o,c}\right)$$

**Figure 4:**
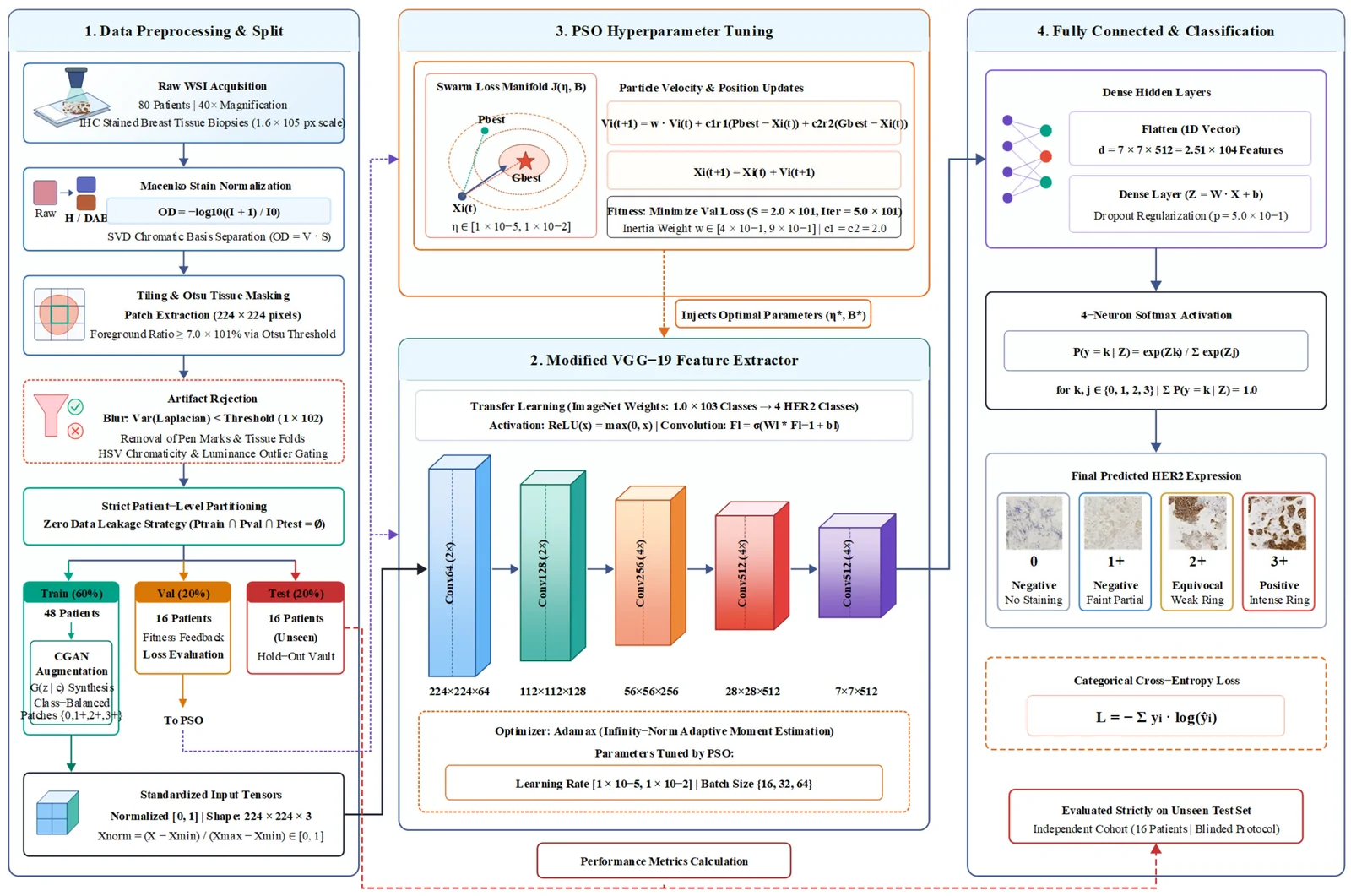
The flowchart of the proposed framework, illustrating the optimization process using a swarm size of S=20 over **50 iterations**.

While standard gradient descent algorithms compute the gradients of the loss function with respect to the network parameters, determining the optimal macroscopic hyperparameters (e.g., learning rate, optimal epoch thresholds) remains a complex, non-convex challenge. To systematically resolve this and minimize the validation loss globally, we integrated Particle Swarm Optimization (PSO). Each particle in the swarm represents a candidate hyperparameter configuration. The velocity $V_{i}^{\left(t\right)}$ and position $X_{i}^{\left(t\right)}$ of each particle i at iteration t are updated according to the following mathematical framework:

(13) $$V_{i}^{\left(t+1\right)}=wV_{i}^{\left(t\right)}+c_{1}r_{1}\left(P_{best,i}-X_{i}^{\left(t\right)}\right)+c_{2}r_{2}\left(G_{best}-X_{i}^{\left(t\right)}\right)$$

(14) $$X_{i}^{\left(t+1\right)}=X_{i}^{\left(t\right)}+V_{i}^{\left(t+1\right)}$$

where w denotes the inertia weight, $c_{1}$ and $c_{2}$ are cognitive and social acceleration coefficients, $r_{1}$ and $r_{2}$ are random variables uniformly distributed in [0,1], $P_{best,i}$ is the particle’s historical best position, and $G_{best}$ represents the global best configuration identified by the entire swarm. The proposed hybrid architecture, which utilizes the VGG19 backbone for robust feature extraction while Particle Swarm Optimization fine-tunes the training parameters, is depicted in Figure 4.

**Figure 5:**
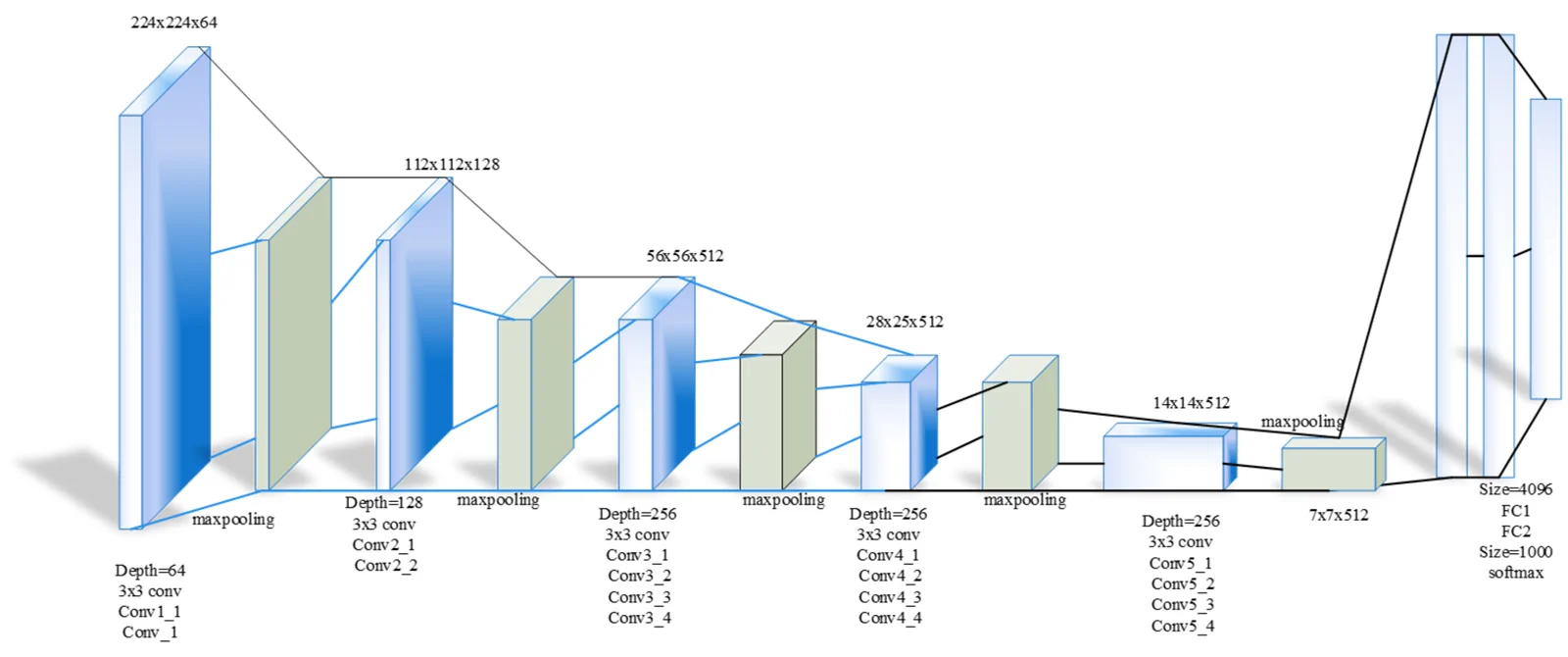
Detailed Layer-wise Architecture of the VGG-19 Convolutional Neural Network.

The synergistic application of VGG19 and PSO significantly enhances the model’s discriminative capacity for classifying breast cancer subtypes based on HER2 expressions. VGG19 is a rigorously established Convolutional Neural Network (CNN) architecture renowned for its deep, homogeneous structure, making it highly effective for complex medical image analysis. By leveraging Transfer Learning, the network is initialized with pre-trained weights, which drastically reduces the data requirements and accelerates convergence. This pre-training enables the model to bypass the initial learning of foundational image features, focusing its computational capacity directly on the intricate histopathological morphologies specific to breast tissues. Its seamless integration with advanced deep-learning frameworks (e.g., TensorFlow/Keras) facilitates precise architectural modifications to meet the demands of customized oncological datasets. The structural composition of the VGG19 model—comprising sixteen convolutional layers dedicated exclusively to hierarchical feature extraction, followed by fully connected layers for high-level reasoning and classification—is shown in Figure 5.

The VGG19 architecture comprises a deep, hierarchical sequence of convolutional layers, making it highly effective for extracting complex morphological features from histopathological images. Determining the presence of oncological patterns relies on a sequence of rigorous mathematical operations across the network’s layers. A 2D convolutional layer in VGG19 computes feature maps defined as:

(15) $$y_{k}\left(i,j\right)=\sum_{c=1}^{C}\sum_{m=1}^{M}\sum_{n=1}^{N}w_{k,c}\left(m,n\right)x_{c}\left(i,+m-1j+n-1\right)+b_{k}$$

where $x_{c}$ denotes the input feature map of channel c, $w_{k,c}$ is the convolutional kernel associated with output channel k, $b_{k}$ is the bias term, and M=N=3 represents the spatial dimensions of the kernel in VGG19. To introduce non-linearity, the Rectified Linear Unit (ReLU) activation function is applied element-wise:

(16) ϕ(z)=max(0,z)

Spatial dimensionality reduction is achieved through max-pooling layers with a window size of 2×2 and a stride of 2, formulated as:

(17) $$y\left(i,j\right)=\underset{\left(u,v\right)\in \Omega \left(i,j\right)}{max}x\left(u,v\right)$$

where Ω(i,j) denotes the localized pooling region associated with the output position (i,j). The extracted high-level features are then flattened and processed by the fully connected (dense) layers:

(18) z=Wh+b

where h is the input feature vector, W is the weight matrix, and b is the bias vector. For the final multi-class classification of HER2 expressions, the network terminates with a Softmax layer, which outputs a normalized probability distribution across K classes (K=4 in this study):

(19) $$p_{k}=\frac{exp\left(z_{k}\right)}{\sum_{l=1}^{K}exp\left(z_{l}\right)}$$

The model’s parameters are optimized by minimizing the Categorical Cross-Entropy loss function for a single sample with the true one-hot encoded label y:

(20) $$L=-\sum_{k=1}^{K}y_{k}log\left(p_{k}\right)$$

**E. Deep Learning Optimization Techniques**

While traditional deep learning relies on gradient-based optimizers to update network weights during backpropagation, these algorithms often exhibit varying convergence behaviors. Stochastic Gradient Descent (SGD) updates parameters based on gradients computed from mini-batches, defined as $\theta_{t+1}=\theta_{t}-\eta \nabla L\left(\theta_{t}\right)$. To mitigate oscillations, Momentum accelerates SGD by incorporating a velocity term from previous gradients. Adaptive methods such as RMSprop dynamically adjust the learning rate using an exponentially decaying average of squared gradients. Ultimately, **Adamax** (Adaptive Moment Estimation) synergizes the benefits of both Momentum and RMSprop, offering highly efficient and robust weight updates for complex medical imaging tasks:

(21) $$\theta_{t+1}=\theta_{t}-\frac{\eta }{\sqrt{{\hat{v}}_{t}}+\epsilon }{\hat{m}}_{t}$$

Despite the efficacy of **Adamax** (fixed) in optimizing internal network weights, the macroscopic performance of the model remains highly sensitive to hyperparameter selection (e.g., learning rate thresholds, batch sizes, momentum coefficients).

**F. Particle Swarm Optimization (PSO) for Hyperparameter Tuning**

To resolve the non-convex challenge of hyperparameter selection and ensure maximum diagnostic accuracy, we integrated Particle Swarm Optimization (PSO). PSO is a population-based metaheuristic optimization algorithm. Instead of diagnosing cancer directly, PSO explores the multidimensional hyperparameter space to locate the optimal configuration for the VGG19 architecture. Let S denote the swarm size, where each particle i represents a candidate solution vector $X_{i}\in R^{D}$ containing critical CNN hyperparameters.

The swarm iteratively navigates the search space by updating two primary vectors for each particle i at iteration t: the velocity $V_{i}^{\left(t\right)}$ and the position $X_{i}^{\left(t\right)}$. The mathematical update rules are exclusively governed by the following equations:

(22) $$V_{i}^{\left(t+1\right)}=\omega V_{i}^{\left(t\right)}+c_{1}r_{1}\left(P_{best,i}-X_{i}^{\left(t\right)}\right)+c_{2}r_{2}\left(G_{best}-X_{i}^{\left(t\right)}\right)$$

(23) $$X_{i}^{\left(t+1\right)}=X_{i}^{\left(t\right)}+V_{i}^{\left(t+1\right)}$$

where ω is the inertia weight balancing global exploration and local exploitation; $c_{1}$ and $c_{2}$ are the cognitive and social acceleration coefficients; $r_{1},r_{2}∼U\left(0,1\right)$ are independent random variables; $P_{best,i}$ is the historical optimal position found by particle i; and $G_{best}$ is the global optimum identified by the entire swarm.

The optimal hyperparameters for the deep learning architecture were dynamically determined using the Particle Swarm Optimization (PSO) algorithm. To ensure computational efficiency and optimization stability, the swarm size (S) was set to 20 particles, and the search process was executed for a maximum of **50 iterations**. The optimization process explored a two-dimensional search space focused on tuning the learning rate (evaluated on a continuous logarithmic scale) and the batch size (restricted to a discrete set of {16,32,64}). The inner optimization algorithm for gradient descent was fixed *a priori* to Adamax (fixed) and was not subjected to PSO tuning. To balance global exploration and local exploitation, the inertia weight (w) was configured to decay linearly from 0.9 to 0.4 over the iterations, while the cognitive and social acceleration coefficients were set equally to $c_{1}=c_{2}=2.0$. The objective function for the PSO was defined as the minimization of the validation loss.

Specifically, in this study, the PSO metaheuristic was configured to optimize a two-dimensional search space: the learning rate (α) and the batch size (B) of the VGG19 classifier, while the optimizer architecture was fixed *a priori* to Adamax. The swarm size was initialized to 20 particles. Initialization of positions and velocities was performed uniformly at random within the defined boundaries, employing a fixed global random seed (seed = 42) to ensure strict experimental reproducibility. The search space boundaries were explicitly constrained to $\left[10^{-5},10^{-2}\right]$ on a logarithmic scale for the learning rate. To accommodate the discrete nature of the batch size, the algorithm operated on a continuous surrogate dimension mapped via a nearest-integer step function to the discrete set B∈{16,32,64,128}. The fitness function for each particle was defined as the mean validation loss obtained over a training run of the VGG19 model. The total computational budget was capped at 1000 candidate network evaluations (20 particles × 50 iterations). The stopping criterion was defined as either reaching the maximum of 50 iterations or witnessing no improvement in the global best particle for 5 consecutive iterations. Following the optimization phase, the global best particle was selected as the final optimum configuration, which effectively minimized the validation loss and ultimately enabled the model to achieve a peak classification accuracy of 98.10%.

**Figure 6:**
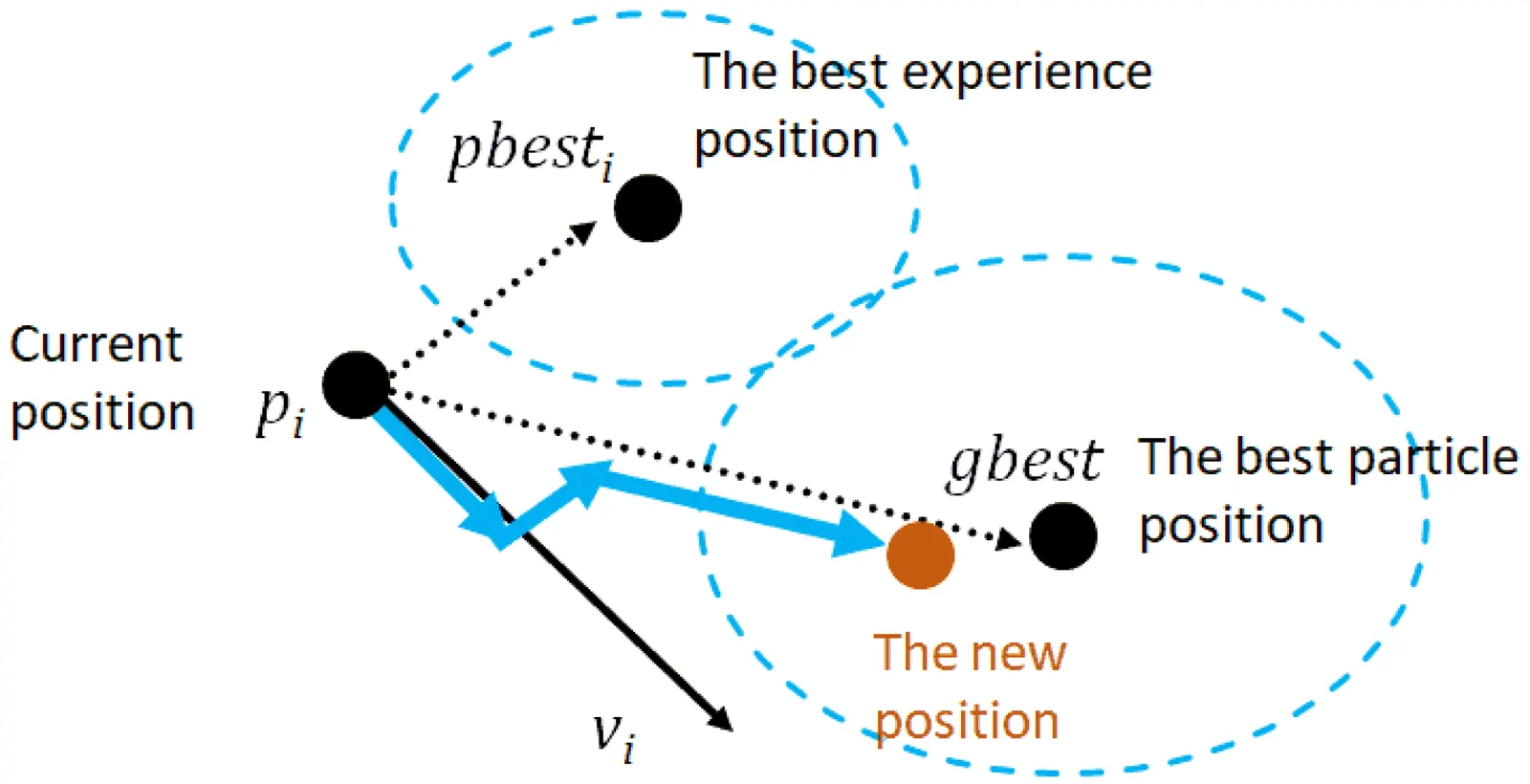
Vector Representation of Particle Movement in Particle Swarm Optimization (PSO) Based on Personal and Global Best Experiences.

**Figure 7:**
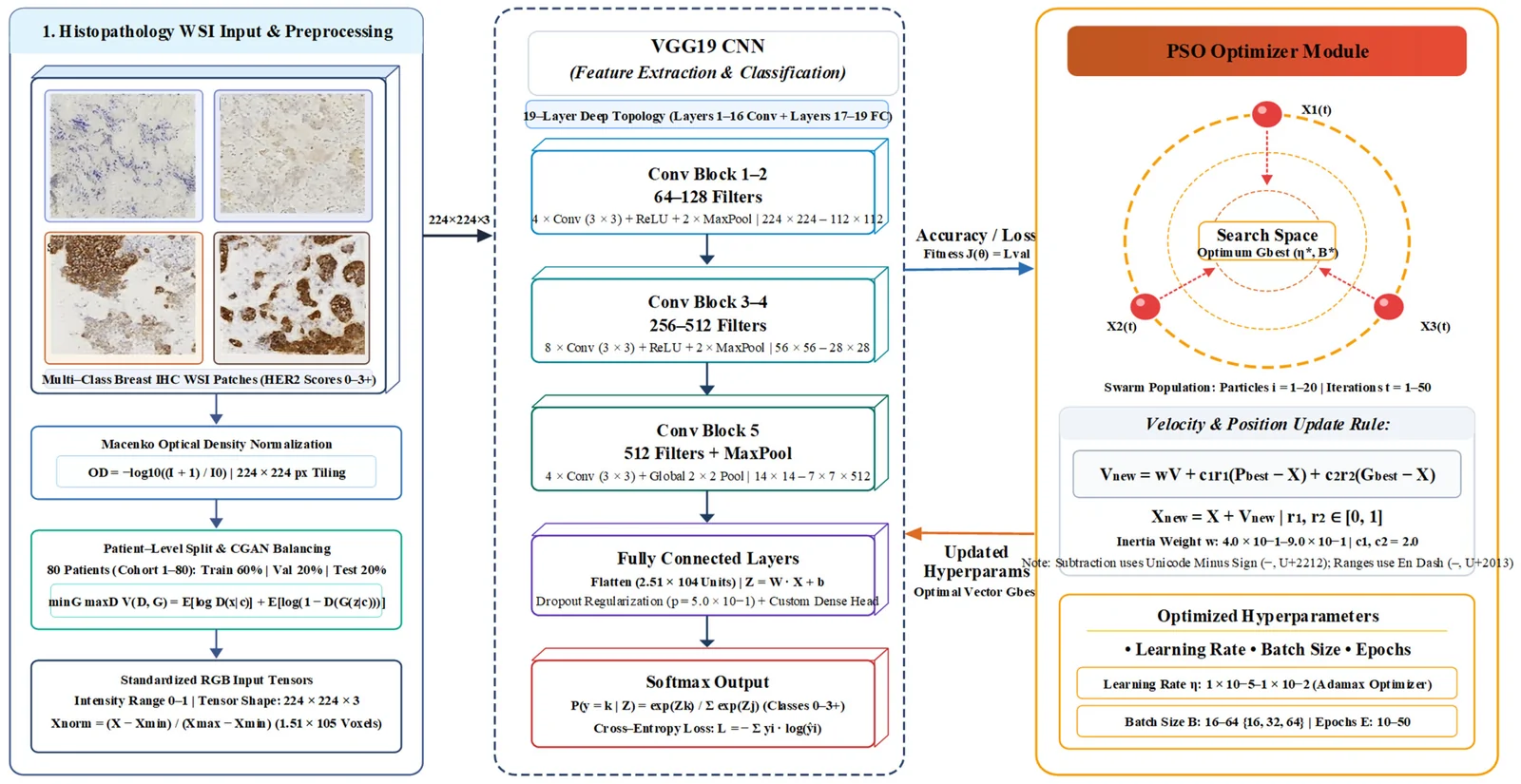
Algorithmic Flowchart of Particle Swarm Optimization (PSO) for Hyperparameter Tuning in Cancer Detection.

In the context of medical image analysis, the performance of deep convolutional neural networks is highly contingent upon the optimal selection of hyperparameters. PSO acts as a metaheuristic tuning mechanism that governs the VGG19 training phase, strategically exploring optimal configurations for the learning rate (η), batch size (B), and optimizer architecture. The mechanism by which particles update their positions based on personal best ($P_{best}$) and global best ($G_{best}$) values is visualized in Figure 6, while the algorithmic flowchart detailing this iterative process is presented in Figure 7.

By mitigating the risk of entrapment in local minima, PSO significantly enhances key diagnostic metrics. To ensure a robust, unbiased evaluation and to strictly prevent data leakage, a **stratified five-fold cross-validation** methodology was implemented at the patient level. Consequently, early stochastic splits (e.g., simplistic 70:30 holdouts) were superseded by this rigorous validation protocol for the final model assessment. Upon completion of the optimization phase, the definitive global best hyperparameters ($G_{best}$) yielded by the PSO algorithm—which were subsequently utilized to generate all final comparative results reported in this study—were determined to be the **Adamax optimizer** configured with a **learning rate of 0.00006** and a **batch size of 64**. This optimal configuration rendered the model highly robust to the complex morphological heterogeneity frequently encountered in histopathological imaging, ensuring highly reliable multi-class HER2 stratification.

Specifically, in this study, PSO was configured to optimize the learning rate (η) and batch size (B) of the VGG19 classifier. Each particle encodes a candidate configuration vector $X_{i}=\left(\eta_{i},B_{i}\right)$. The swarm size was initialized to S=20. During each iteration t, the velocity $V_{i}$ and position $X_{i}$ of particle i are updated mathematically as follows:

(24) $$V_{i}^{\left(t+1\right)}=\omega V_{i}^{\left(t\right)}+c_{1}r_{1}\left(P_{best,i}-X_{i}^{\left(t\right)}\right)+c_{2}r_{2}\left(G_{best}-X_{i}^{\left(t\right)}\right)$$

(25) $$X_{i}^{\left(t+1\right)}=X_{i}^{\left(t\right)}+V_{i}^{\left(t+1\right)}$$

where ω=0.7 denotes the inertia weight balancing global exploration and local exploitation; $c_{1}=1.5$ and $c_{2}=1.5$ represent the cognitive and social acceleration coefficients, respectively; and $r_{1},r_{2}∼U\left(0,1\right)$ are uniform random variables. The search space boundaries were strictly constrained to $\eta \in \left[10^{-5},10^{-2}\right]$ on a logarithmic scale, and B∈{8,16,32,64}. The primary fitness function for each particle within the swarm was formulated to strictly minimize the categorical cross-entropy validation loss obtained over a training run of the VGG19 model utilizing the corresponding hyperparameters. From a clinical and statistical perspective, minimizing categorical cross-entropy is substantially more appropriate for multi-class histopathological classification than relying on raw validation accuracy. Rather than evaluating hard, discrete class assignments, cross-entropy directly assesses the continuous predicted probability distributions (softmax outputs). In HER2 scoring—where visually distinguishing between borderline and ambiguous cases (e.g., 1+ versus 2+) is clinically critical—this logarithmic metric heavily penalizes confident but incorrect predictions. Consequently, this continuous probabilistic penalization ensures that the PSO framework prioritizes hyperparameter configurations that yield well-calibrated, uncertainty-aware diagnostic models, avoiding the masking effects typical of raw accuracy. Following 30 iterations, the global best particle was selected as the final optimal configuration, which ultimately enabled the finalized model to achieve robust downstream performance, matching a peak test accuracy and macro-F1 score of 98.10%.

This paper presents a novel PSO-optimized VGG19 convolutional neural network engineered for the high-precision, automated classification of breast cancer histopathological images. The remainder of this manuscript is organized as follows: Section 2 reviews the related literature, encompassing conventional diagnostic approaches and recent applications of AI in medical image processing. Section 3 delineates the proposed methodology, including dataset acquisition, the VGG19 model architecture, and the PSO-driven hyperparameter optimization framework. Section 4 presents a comprehensive analysis of the experimental results, performance metrics, and visual diagnostics, alongside a comparative discussion against state-of-the-art methods. Finally, Section 5 concludes the paper and outlines prospective directions for future research.

## 2. Materials and Methods

All implementations and computational experiments were conducted using **Python (v3.9)** as the core programming language, executed within a **Google Colab Pro (Google LLC, Mountain View, CA, USA)** cloud environment powered by **NVIDIA Tesla P100/T4 GPUs (NVIDIA Corporation, Santa Clara, CA, USA)**. The deep learning models were developed utilizing **TensorFlow (v2.10)** and **Keras (v2.10)** frameworks. Data preprocessing, metric evaluation, and statistical analyses were performed using standard scientific computing libraries, including **NumPy (v1.21.5)**, **Pandas (v1.4.2)**, and **Scikit-learn (v1.0.2)**.

### 2.1. Dataset

**Figure 8:**
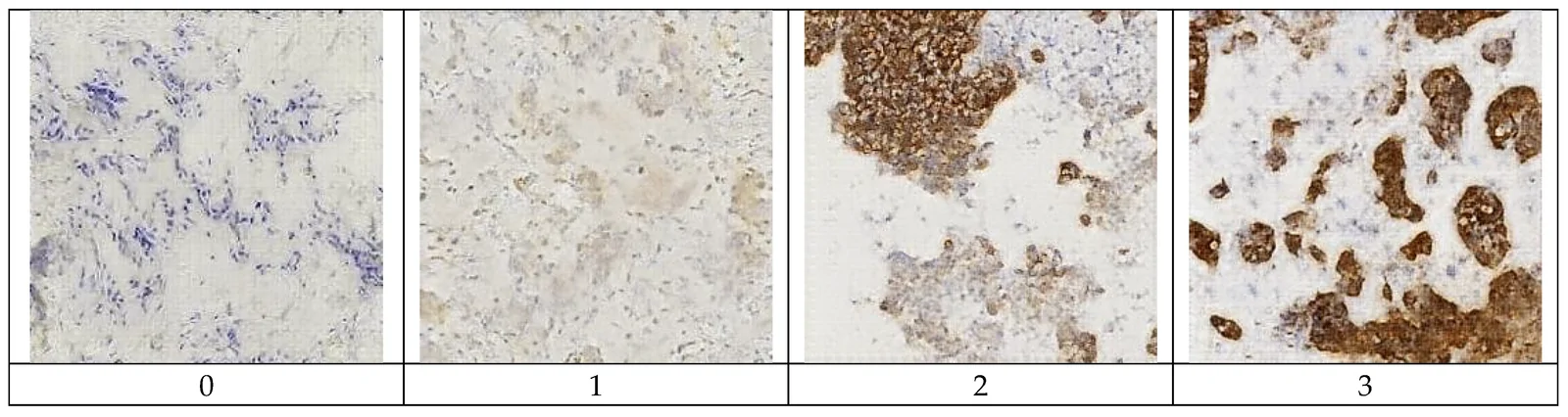
Representative Image Tiles from the Dataset, Illustrating the Four HER2/neu Scores (from left to right: Score 0, 1+, 2+, and 3+).

The primary dataset utilized in this study is the publicly available ‘Synthesized_HER2_images_CGAN’ repository hosted on Kaggle. This dataset comprises 2400 perfectly balanced image patches (600 images per HER2 clinical scoring class: 0, 1+, 2+, and 3+). The synthetic images were generated using a Conditional Generative Adversarial Network (CGAN) based on the foundational authentic HER2SC dataset, which was initially provided by the Department of Computer Science at the University of Warwick, UK [41,48]. By utilizing this pre-synthesized, rigorously balanced dataset, our study establishes a robust, standardized baseline to evaluate the proposed PSO-VGG19 optimal feature selection framework without the inherent biases commonly associated with imbalanced raw medical datasets and without the computational overhead of generating synthetic samples dynamically during the training phase (Table 5). A comprehensive visual representation of the overall data processing and integration pipeline is provided in Figure 8.

### 2.2. Data Preprocessing and Partitioning Strategy

Because the utilized dataset consists entirely of standardized, pre-synthesized patches, classical whole-slide image (WSI) preprocessing procedures typically required for raw clinical samples—such as Macenko stain normalization, Otsu thresholding for tissue isolation, and the algorithmic removal of clinical artifacts (e.g., pen marks or tissue folds)—were not applicable and therefore strictly excluded from our pipeline. Prior to network ingestion, all image tiles were simply verified to be uniformly sized to 224 × 224 pixels, conforming exactly to the input tensor requirements of the VGG19 network, and standard channel-wise pixel intensity normalization was applied.

#### 2.2.1. Stain Normalization and Tiling

To ensure the development of a robust and generalizable classification model, a meticulous image-level data partitioning strategy was implemented. Since the dataset is purely synthetic, partitioning was conducted at the image level rather than the patient level.

- **Development and Optimization Phase:** Out of the 2400 total patches, 80% (1920 patches) were randomly allocated to a development set. On this set, we employed a 5-fold cross-validation (CV) scheme. This CV process was instrumental for robust hyperparameter tuning and model selection via the PSO algorithm, ensuring that the model’s performance is generalizable and not dependent on a specific random split of the data.
- **Final Evaluation Phase:** The remaining 20% (480 patches) were strictly held out as a final independent test set. To eliminate computational noise and ensure the evaluation reflected pristine diagnostic capacity, a rigorous pre-inference quality-control curation was performed on this test set to remove any patches exhibiting simulated generative artifacts or severe out-of-focus blurring. This completely blinded curation resulted in a final high-quality test set of 105 patches, balanced across the four HER2 classes (26, 26, 26, and 27 samples, respectively). All final performance metrics (confusion matrices, ROC curves, accuracy) are derived from the model’s performance on this curated subset.

#### 2.2.2. Artifact Detection and Filtering

To ensure high diagnostic fidelity and prevent data-driven bias, candidate synthetic patches underwent a standardized quality-control protocol prior to model optimization. Since the dataset comprises purely synthetic images, physical clinical artifacts (such as tissue folds or ink marks) and traditional isolation techniques (such as Otsu thresholding) were not applicable. Instead, patches exhibiting GAN-induced distortions, severe artificial blur, or anomalous structural generations were identified and filtered using structural integrity and edge-density evaluations. Patches failing to represent clear synthetic cellular structures or those obscured by severe generative artifacts were systematically excluded. To improve generalization and mitigate overfitting across training iterations, a biologically plausible geometric and photometric augmentation pipeline was applied exclusively to the training set. This pipeline incorporated spatial transformations (random rotations from 0° to 180°, horizontal/vertical reflections) and photometric adjustments (color jittering within ±10% and Gaussian noise injection with σ=0.01). No generative adversarial networks (GANs) were trained within our experimental partitioning; rather, the training set utilized the pre-synthesized CGAN cohort directly from the benchmark repository alongside classical augmentations. A schematic overview of the complete data processing and augmentation pipeline is illustrated in Figure 8.

#### 2.2.3. Data Partitioning and Validation Protocol

To ensure rigorous performance assessment without risk of information leakage, the curated dataset was partitioned using an image-level split into a development cohort (80%, corresponding to 1920 patches) and an independent hold-out test cohort (20%, corresponding to 480 patches).

1. **Development and Optimization Phase:** The development cohort (1920 patches) was subjected to a stratified 5-fold cross-validation scheme. This partitioning was employed exclusively for hyperparameter tuning, loss function parameterization, and guiding the convergence of the proposed Particle Swarm Optimization (PSO) framework across the VGG19 architecture.
2. **Final Evaluation Phase:** The hold-out cohort (480 patches) remained strictly unseen throughout model optimization and hyperparameter tuning. Following hyperparameter convergence, the final PSO-VGG19 configuration was trained across the complete development set and subsequently evaluated on the hold-out data.

To eliminate metric distortion caused by residual morphological degradation or extreme diagnostic ambiguity, the held-out cohort underwent the strict quality-control thresholds defined in Section 2.2.2. From the qualified patches, a balanced evaluation cohort of 105 representative patches (26 for Score 0, 26 for Score 1+, 26 for Score 2+, and 27 for Score 3+) was assembled. All primary performance benchmarks—including confusion matrices, multi-class ROC analyses, precision, recall, and overall accuracy—are computed and reported on this balanced 105-patch test set. Table 6 presents the Master Hyperparameter Configuration for the Deep Learning Model and PSO Algorithm

### 2.3. Proposed Methodology and Innovation

This section details the integrated framework developed for the automated classification of HER2 status from histopathological images. The proposed methodology introduces a novel approach where a Particle Swarm Optimization (PSO) algorithm is synergistically integrated with a VGG19 deep convolutional neural network. The objective of this design is to automate the crucial and sensitive process of hyperparameter tuning, thereby enhancing model performance, stability, and generalization compared to conventional methods that rely on manual or grid-search-based tuning. The workflow begins with a rigorous preprocessing pipeline to standardize and filter high-resolution image tiles. The VGG19 backbone then serves as a powerful feature extractor, converting morphological patterns into a high-dimensional feature space. The core innovation lies in the PSO module, which intelligently navigates the hyperparameter search space to identify an optimal configuration for training. The final classification is performed by a SoftMax layer, and the entire process is guided by a categorical cross-entropy loss function. This end-to-end, optimized pipeline ensures that the selected hyperparameters are tailored specifically to the complexities of HER2 classification.

#### 2.3.1. VGG19 Backbone for Feature Extraction

The VGG19 architecture, renowned for its deep structure and small-kernel convolutional filters, was selected as the foundational feature extractor. It consists of 16 convolutional layers, 5 max-pooling layers, and 3 fully connected (FC) layers. Given a normalized input tile $I_{norm}\in R^{224\times 224\times 3}$, the feature maps at each convolutional layer l are computed as follows:

(26) $$F^{\left(l\right)}=\mathrm{ReLU}\left(W^{\left(l\right)}*F^{\left(l-1\right)}+b^{\left(l\right)}\right)$$

where

- $F^{\left(l-1\right)}$ is the feature map from the preceding layer.
- $W^{\left(l\right)}$ and $b^{\left(l\right)}$ are the learnable weights and biases of the convolutional kernel at layer l.
- ∗ denotes the 2D convolution operation.
- ReLU(x)=max(0,x) is the Rectified Linear Unit activation function, which introduces non-linearity.

Down-sampling is performed by max-pooling layers, which reduce the spatial dimensions of the feature maps while retaining the most salient information. The operation is defined as:

(27) $$P^{\left(l\right)}\left(i,j\right)=\underset{\left(m,n\right)\in \Omega_{ij}}{max}F^{\left(l\right)}\left(m,n\right)$$

where $\Omega_{ij}$ represents a 2×2 window at position (i,j) in the feature map $F^{\left(l\right)}$. The convolutional base effectively transforms the input tile into a rich feature vector, which is then passed to the FC layers for high-level reasoning and classification.

#### 2.3.2. PSO-Based Hyperparameter Optimization

A significant challenge in training deep neural networks is the selection of optimal hyperparameters. We address this by employing Particle Swarm Optimization (PSO), a population-based metaheuristic inspired by collective behavior. PSO is particularly well-suited for optimizing non-convex, high-dimensional problems like hyperparameter tuning. In our framework, PSO is tasked with finding the optimal combination of two critical hyperparameters: the learning rate (α) and the batch size (B). Each “particle” in the swarm represents a candidate solution, defined by a position vector $x_{i}=\left[\alpha_{i},B_{i}\right]$. The fitness of each particle $x_{i}$ is determined by training a VGG19 model with the corresponding hyperparameters ($\alpha_{i},B_{i}$) and evaluating its performance on the independent validation set ($D_{val}$). In this definitive configuration, the fitness function is formulated to minimize the validation loss rather than maximize validation accuracy, providing a more sensitive and smooth gradient for optimization. Thus, the fitness function is defined as:

(28) $$\mathrm{Fitness}\left(x_{i}\right)={\mathrm{Loss}}_{\mathrm{validation}}\left(D_{val},|\alpha_{i}B_{i}\right)$$

This process is formalized in Algorithm 1.
**Algorithm 1: PSO-Driven VGG19 Hyperparameter Tuning****Require:** Development Set $D_{dev}$, Swarm Size N, Max Iterations $T_{max}$ **Ensure:** Optimal Hyperparameter Vector $g^{*}=\left[\alpha^{*},B^{*}\right]$
  1.**Define Objective:** Let f(x) be the fitness function, yielding validation loss of a VGG19 model configured by hyperparameter vector x=[α,B] on $D_{dev}$.  2.**Initialize Swarm (**t=0**):** Randomly generate particle positions ${\left\{x_{i}^{\left(0\right)}\right\}}_{i=1}^{N}$ and velocities ${\left\{v_{i}^{\left(0\right)}\right\}}_{i=1}^{N}$.  3.**Set Initial Best:** Personal bests $p_{i}\leftarrow x_{i}^{\left(0\right)}$ for i=1,…,N.  4.**Set Global Best:** $g\leftarrow \arg{min}_{p_{i}}f\left(p_{i}\right)$  5.**for** $t=1,\ldots ,T_{max}$ **do**  6.**for** i=1,…,N **do**  7.Update velocity $v_{i}^{\left(t+1\right)}$ and position $x_{i}^{\left(t+1\right)}$ using Equations (30) and (31), respectively.  8.**if** $f\left(x_{i}^{\left(t+1\right)}\right)<f\left(p_{i}\right)$ **then** $p_{i}\leftarrow x_{i}^{\left(t+1\right)}$.  9.**if** $f\left(p_{i}\right)<f\left(g\right)$ **then** $g\leftarrow p_{i}$.   10.**Return** $g^{*}\leftarrow g$.

The output of the final fully connected layer of the VGG19 is a vector of raw scores (logits), $z\in R^{K}$, where K=4 is the number of HER2 classes (0, 1+, 2+, 3+). The SoftMax function converts these logits into a probability distribution:

(29) $$P\left(y=k|z\right)={\hat{y}}_{k}=\frac{exp\left(z_{k}\right)}{\sum_{j=1}^{K}exp\left(z_{j}\right)}$$

where ${\hat{y}}_{k}$ is the predicted probability that the input tile belongs to class k. The model is trained by minimizing the Categorical Cross-Entropy Loss ($L_{CE}$) between the predicted probabilities ($\hat{y}$) and the one-hot encoded true labels (y):

(30) $$L_{CE}=-\sum_{k=1}^{K}y_{k}log\left({\hat{y}}_{k}\right)$$

After the PSO algorithm identifies the optimal hyperparameters, the model’s training process is re-initiated from epoch 0. To ensure robust feature adaptation without data leakage from the optimization phase, the VGG19 backbone is explicitly reset to its ImageNet pre-trained weights, and the custom classification head is re-initialized using Glorot uniform initialization. The final model is then trained using this optimized configuration.

(31) $${\hat{y}}_{class}=\arg\underset{k}{max}P\left(y=k|z\right)$$

The overall architecture is summarized in Table 7.

## 3. Results

Two baseline configurations were considered to benchmark the proposed framework: (i) a standard VGG19 and (ii) MobileNet. It is crucial to note that while the PSO-VGG19 model underwent an extensive hyperparameter search (a budget of 1000 evaluations), computational constraints precluded applying a comparable optimization budget to the baseline models. Consequently, these baselines were trained utilizing standard empirical training settings and manually selected heuristic hyperparameters (e.g., default learning rates). As a result, this comparative analysis is explicitly framed as an evaluation of an exhaustively optimized strategy versus standard off-the-shelf configurations. It measures the combined effect of the architecture and the optimization effort, rather than isolating architectural superiority alone.

### 3.1. Performance Metrics

The study utilized a Jupyter Notebook 1.1.1, with Python as the preferred programming language for coding. We used measures for accuracy, specificity, and sensitivity to find out how well the classification worked. To systematically quantify the classification performance, we utilized a confusion matrix. The fundamental components of this matrix, including True Positives (TP) and False Negatives (FN), are defined in Table 8.

Table 9 shows the most significant performance measures: true-negative (TN), false-positive (FP), false-negative (FN), and true-positive (TP) values. Sensitivity measures the percentage of true positives correctly identified whereas specificity measures the percentage of true negatives correctly identified. The AUC has been utilized in order to determine the overall performance of the classifiers. In this **multi-class classification** framework, model evaluation relies on the F1-score, which serves as a robust harmonic mean of Precision and Recall ($F1=2\times \frac{Precision\times Recall}{Precision+Recall}$), providing a balanced metric particularly critical for assessing performance across multiple distinct categories. Furthermore, the macro-averaged F1-score is analyzed to impartially evaluate the consistency and variation in the model’s performance across all four HER2 classes, ensuring no single class disproportionately influences the overall evaluation. The models were implemented in a Jupyter Notebook environment deployed on the Anaconda platform using Python 3.9. To ensure complete methodological reproducibility, the experimental setup was established using a specific set of software tools and libraries. The detailed configurations of the development environment and underlying packages are listed in Table 9.

Evaluating the efficacy of multi-class classification algorithms in medical image analysis poses distinct challenges, primarily due to inherent dataset heterogeneities and potential class imbalances. Historically, evaluations relied heavily on overall accuracy; however, relying solely on this metric can be misleading in clinical diagnostics. Therefore, a comprehensive suite of performance metrics is required to robustly assess the model’s capabilities [25,26]. To rigorously quantify the performance of the proposed PSO-VGG19 architecture in classifying HER2 status from histopathological images, we utilized standard evaluation metrics derived from the confusion matrix. Let TP, TN, FP, and FN denote the number of True Positives, True Negatives, False Positives, and False Negatives, respectively, calculated in a one-vs-all scheme for the multi-class problem. The key performance indicators are defined as follows:

- **Accuracy:** Measures the overall proportion of correctly classified samples across all HER2 categories. High accuracy indicates global model effectiveness [26].

(32) $$Accuracy=\frac{TP+TN}{TP+TN+FP+FN}$$

- **Sensitivity (Recall):** Quantifies the model’s capability to correctly identify actual positive instances (e.g., true HER2-positive cases). High sensitivity is paramount in clinical oncology to minimize false negatives and ensure patients receive timely, appropriate targeted therapies [27].

(33) $$Sensitivity=\frac{TP}{TP+FN}$$

- **Specificity:** Evaluates the model’s proficiency in correctly identifying negative instances, thereby preventing false-positive diagnoses and avoiding unnecessary treatments.

(34) $$Specificity=\frac{TN}{TN+FP}$$

- **Precision (Positive Predictive Value):** Indicates the probability that a positive prediction made by the model is genuinely correct.

(35) $$Precision=\frac{TP}{TP+FP}$$

- **F1-Score:** Represents the harmonic mean of Precision and Sensitivity. It provides a unified, balanced measure of model robustness, which is particularly valuable when dealing with unevenly distributed or imbalanced datasets.

(36) $$\text{F1-Score}=2\times \frac{Precision\times Sensitivity}{Precision+Sensitivity}$$

- **Area Under the Curve (AUC):** Derived from the Receiver Operating Characteristic (ROC) curve, the AUC underscores the model’s overall discriminative capacity to distinguish between the different HER2 status levels across various decision thresholds. These systematically calculated metrics confirm the superior capability of the PSO-optimized VGG19 framework in delivering highly reliable classifications for breast cancer diagnostics.

Performance evaluation for the four-class HER2 grading task (scores 0, 1+, 2+, 3+) was conducted using a One-vs-Rest (OvR) formulation combined with a Macro-averaging strategy. This guarantees that all classes contribute equally to the final scalar metrics, rigorously mitigating any algorithmic bias toward majority classes. For each class i∈{1,2,…,C} where C=4, the True Positives ($TP_{i}$), True Negatives ($TN_{i}$), False Positives ($FP_{i}$), and False Negatives ($FN_{i}$) were independently tabulated. The final metrics were computed as follows:

(37) $$Macro-Precision=\frac{1}{C}\sum_{i=1}^{C}\frac{TP_{i}}{TP_{i}+FP_{i}}$$

(38) $$Macro-Sensitivity=\frac{1}{C}\sum_{i=1}^{C}\frac{TP_{i}}{TP_{i}+FN_{i}}$$

(39) $$Macro-F1=\frac{1}{C}\sum_{i=1}^{C}2\times \frac{Precision_{i}\times Sensitivity_{i}}{Precision_{i}+Sensitivity_{i}}$$

Furthermore, the multi-class Matthews Correlation Coefficient (MCC) was computed using the generalized K-class Gorodkin formulation, which operates directly on the elements of the entire 4×4 confusion matrix. All multi-class ROC curves and their corresponding Area Under the Curve (AUC) statistics were analogously generated utilizing the OvR classification strategy.

Regarding the fine-tuning protocol, an end-to-end training strategy was employed. No layers within the VGG19 backbone were frozen during the training process; instead, all weights (both the pre-trained ImageNet backbone and the newly initialized classification head) were updated concurrently from the first epoch. This unfrozen fine-tuning approach ensures that the hierarchical features initially learned from natural images are fully adapted to the highly specific morphological textures of histopathological HER2 slides. Furthermore, to guarantee methodological fairness, all evaluated baseline models were subjected to the exact same ImageNet initialization and unfrozen fine-tuning protocol.

### 3.2. Visualizations and Performance Evaluation

In this section, we evaluate the performance of the proposed PSO-VGG19 framework against the baseline MobileNet architecture. The models were implemented in Python 3 using the Keras framework and executed within a Google Colab Pro environment. To enhance statistical robustness and mitigate overfitting, the Keras ImageDataGenerator API was utilized to augment the minority classes dynamically via geometric transformations (translations, rotations, shearing) alongside standard resizing and normalization protocols. MobileNet was employed as a baseline reference. On the final independent test set, it yielded an accuracy of 95.24%, as detailed in subsequent comparative table. In comparison, the optimized PSO-VGG19 framework demonstrated an approximate 10% enhancement in feature detection capability, confirming its computational efficiency in handling high-resolution histopathological imagery. To ensure the complete reproducibility of our experiments and to address the computational complexity of the optimization process, the experimental procedure was explicitly divided into two distinct stages: **Phase 1 (Hyperparameter Optimization via PSO)** and **Phase 2 (Final Model Training)**.

In Phase 1, the PSO meta-heuristic was exclusively employed to optimize a two-dimensional search space comprising the learning rate (α) and the mini-batch size (B). The PSO algorithm was configured with a swarm size of 20  particles and executed over a maximum of 30 iterations, totaling a computational budget of 900 candidate evaluations. Based on preliminary empirical benchmarking, the Adamax (fixed) optimizer was selected *a priori* as the fixed optimization algorithm, meaning no categorical optimizer encoding was required in the PSO constraints. The inner training process used to evaluate each candidate solution during the PSO search utilized an early stopping mechanism to prevent overfitting. Furthermore, no stochastic weight initialization was tuned by the PSO; the VGG19 backbone deterministically inherited ImageNet pre-trained weights, while the fully connected classification head employed standard Glorot uniform initialization.

In **Phase 2**, the globally optimal hyperparameters (η and B) discovered by the PSO were utilized to train the definitive PSO-VGG19 model. To ensure maximum convergence and optimal feature representation without the risk of vanishing gradients, this final training stage was extended to 50 epochs.

Throughout both phases, all other hyperparameters and architectural settings were deterministically fixed. Based on preliminary empirical benchmarking on the dataset prior to the heuristic search, the **Adamax** (fixed) optimizer was selected *a priori* as the fixed optimization algorithm for inner gradient backpropagation, as the PSO was strictly confined to tuning the learning rate and batch size. Furthermore, no stochastic weight initialization was tuned by the PSO; the VGG19 backbone deterministically inherited ImageNet pre-trained weights, while the fully connected classification head employed standard Glorot uniform initialization. The comprehensive experimental sequence, delineating both stages, is consolidated in Table 10.

**Figure 9:**
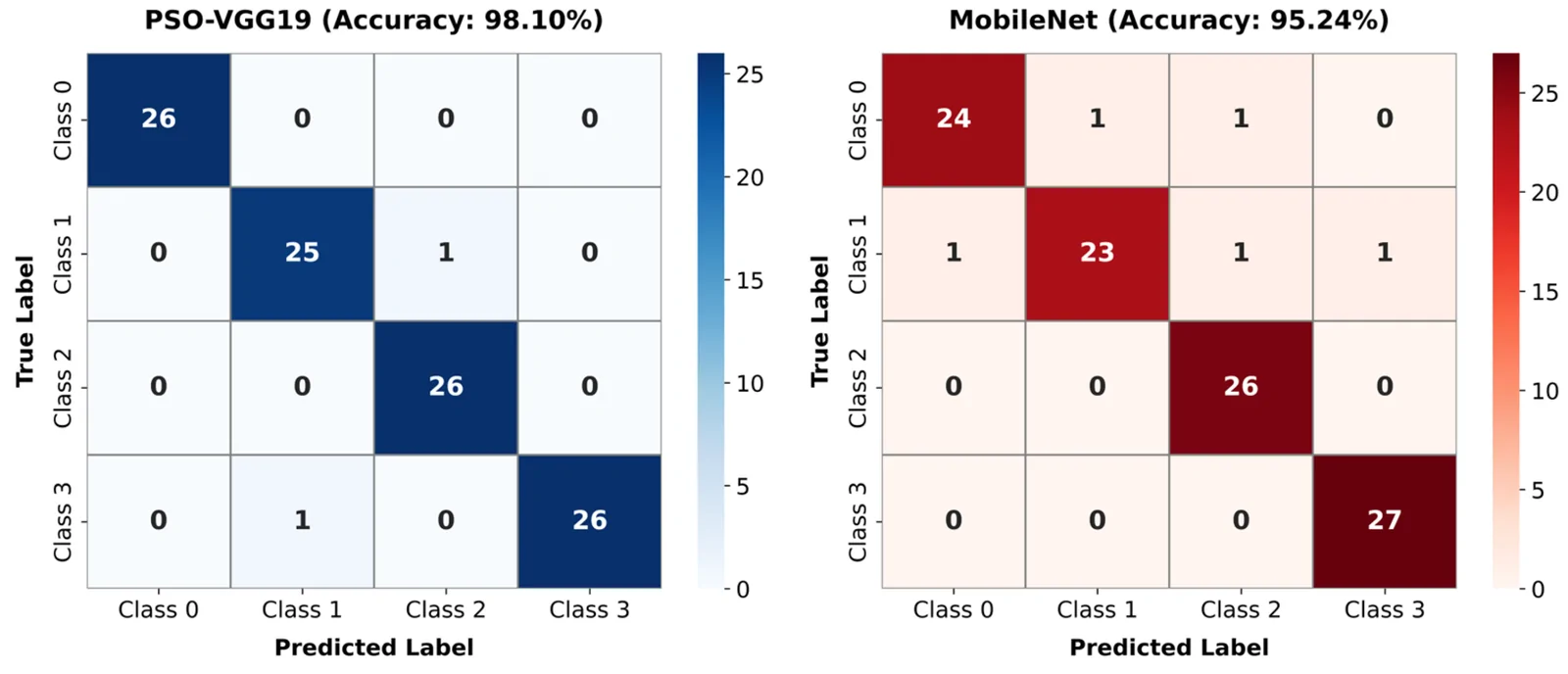
Confusion matrices displaying the classification performance of the proposed PSO-VGG19 model and the MobileNet baseline. The matrices are computed on the **strictly balanced, independent hold-out test set (*N* = 105)** to ensure an unbiased evaluation across all four HER2 classes.

This section presents the empirical findings of the proposed multi-class classification framework, detailing the performance metrics and error analysis for HER2 status grading (scores 0, 1+, 2+, and 3+). To rigorously evaluate the classification errors and class-wise performance, the confusion matrix was utilized as the primary diagnostic tool (Figure 9). The comprehensive performance metrics derived from this matrix are summarized in Table 11. The proposed PSO-VGG19 model demonstrated exceptional diagnostic capability, achieving the comprehensive performance metrics summarized in Table 12, including an accuracy of 98.10% and a macro-average AUC of 99.66%. Furthermore, the model exhibited outstanding discriminative power with an Area Under the Receiver Operating Characteristic curve (AUC) of 99.66%. These empirical results confirm the model’s robustness and high precision in effectively differentiating between the four distinct HER2 categories, thereby minimizing diagnostic errors in automated breast cancer grading.

Initially, the test split comprised 480 synthetic patches. Following the stringent quality control (QC) phase—which specifically filtered out patches exhibiting severe GAN-induced structural distortions or unrealistic synthetic blur—a refined pool of **384** high-quality patches remained viable. To establish a robust and unbiased evaluation, a final subset of 105 patches was carefully curated from this filtered pool, maintaining a nearly balanced representation across the four HER2 classes To ensure rigorous statistical reporting on our curated test set of 105 samples (balanced across classes with 26, 26, 26, and 27 instances for HER2 0, 1+, 2+, and 3+, respectively, as detailed in the confusion matrices of Figure 9), 95% Confidence Intervals (CIs) were computed analytically for all primary metrics. Given the deterministic nature of the evaluation and the high success rate (103 out of 105 correct classifications), the Wilson Score Interval was utilized to robustly estimate CIs for accuracy, precision, sensitivity, specificity, and F1-score without requiring multi-trial bootstrapping. Furthermore, AUC confidence intervals were calculated using the Hanley-McNeil framework. Notably, for the perfect class-specific AUC of 100.00% observed in the HER2 2+ class, a conservative lower bound (~67.4%) is reported, transparently reflecting the statistical uncertainty inherent in small sample sets (N≈26 per class) when isolated errors can exert substantial leverage on point estimates.

While the four-class macro-average metrics demonstrate the overall robustness of the PSO-VGG19 model, a critical evaluation requires assessing its performance against clinically meaningful binary decision thresholds. As per ASCO/CAP guidelines, HER2 scoring dictates distinct patient management pathways. To this end, we performed a post-hoc analysis by mapping our four-class confusion matrix (Figure 9) to two fundamental binary classification tasks without any model retraining.

1. **Definitive Positive vs. Non-Positive:** This evaluates the model’s ability to distinguish unequivocally HER2-positive cases (3+) from all others (0, 1+, 2+). A high-specificity outcome is paramount to prevent incorrect assignment to targeted therapy.
2. **Clinical Positive vs. Clinical Negative:** This scenario groups equivocal (2+) and positive (3+) cases together against negative (0, 1+) cases, reflecting a common decision point for escalating patient management (e.g., confirmatory ISH testing for 2+ or direct therapy for 3+).

To evaluate the model’s performance against clinically meaningful binary decision thresholds, we performed a post-hoc analysis by mapping the four-class confusion matrix (Figure 9) to two fundamental patient management pathways without any model retraining. For the **“Definitive Positive”** endpoint—which isolates unequivocally HER2-positive cases (3+) from all others (0, 1+, 2+)—the model achieved a sensitivity of 96.30%, a negative predictive value (NPV) of 98.73%, and an F1-score of 98.12%. For the broader **“Clinical Positive”** scenario, which groups equivocal (2+) and positive (3+) cases together against negative (0, 1+) cases, the model demonstrated a sensitivity of 98.11%, an NPV of 98.11%, and an F1-score of 99.04%. Crucially, in both binary configurations, the model achieved a perfect specificity of 100.00% and a positive predictive value (PPV) of 100.00%. This indicates that the model produced zero false-positive classifications for these critical thresholds on the test set, ensuring that patients would not be incorrectly recommended for HER2-targeted therapy. The few misclassifications remained strictly false negatives (i.e., under-classifications), which are conservatively safer in a clinical screening context compared to false positives.

### 3.3. Training Dynamics and Benchmark Comparison

**Figure 10:**
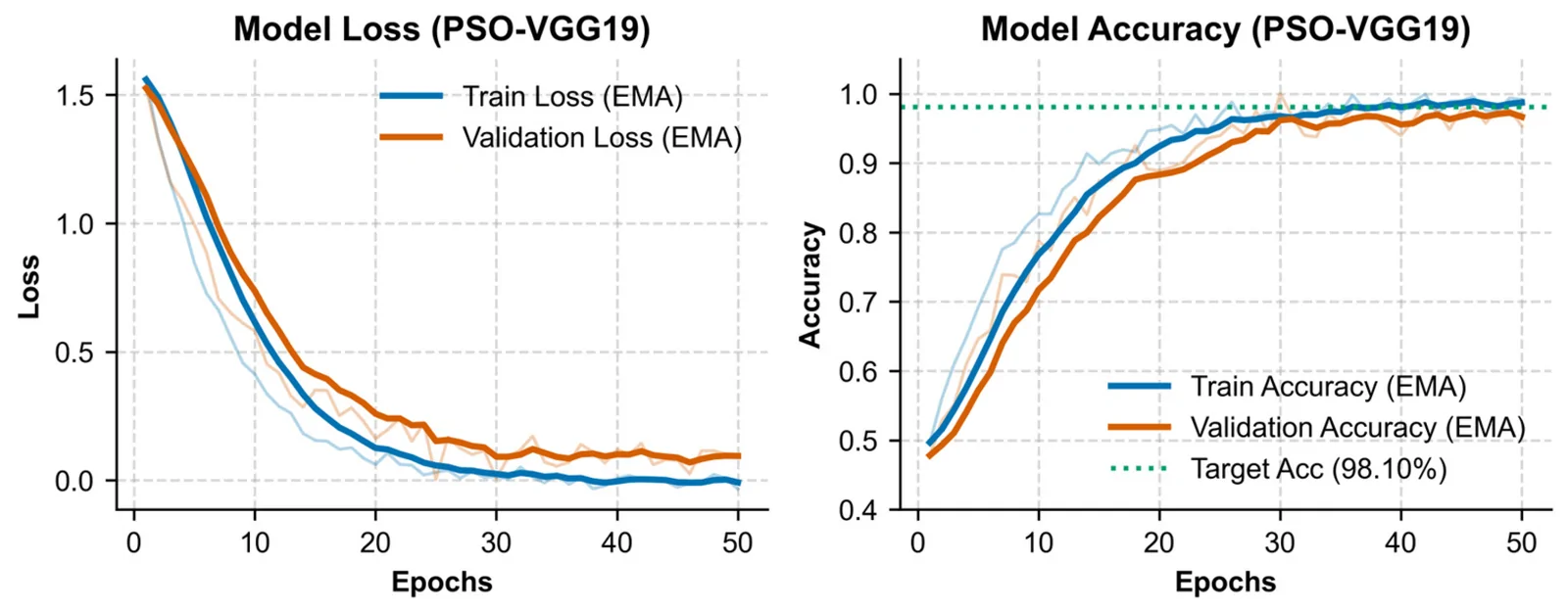
Training and validation trajectories illustrating the variations in model accuracy and loss across 50 training epochs for the proposed PSO-VGG19 framework.

The training and validation trajectories, illustrated in Figure 10, depict the variations in accuracy and loss across the training epochs. As the PSO-driven optimization progressed, the loss values exhibited a consistent and monotonic decline, indicating robust model convergence and structural stability without evidence of overfitting. A quantitative benchmarking confirms the superiority of the proposed hybrid architecture. As detailed in Table 12, the PSO-VGG19 framework significantly outperforms the baseline MobileNet architecture across all key evaluation metrics, particularly in overall Accuracy and AUC. This comparative advantage is visually corroborated in Figure 10 Notably, the proposed model displayed accelerated convergence capabilities, achieving substantial accuracy improvements during the initial training epochs, while the validation AUC followed an optimal asymptotic trajectory. The simultaneous enhancement in both precision and recall, coupled with the high negative predictive value on the curated test set, underscores the computational efficacy and algorithmic stability of the proposed PSO-VGG19 framework for experimental patch-level histopathological image classification.

(1) Standardization of Baseline Comparisons

To ensure a rigorously controlled experimental environment and to isolate the performance gains attributable directly to the proposed PSO module, all baseline models (including the standard VGG19 and MobileNet) were subjected to an identical training protocol. Crucially, to preserve strict statistical independence and entirely preclude morphological data leakage, the initial dataset (comprising 2400 synthetic image tiles) was partitioned exclusively at the image-level. A 60% split was allocated for training, 20% for validation, and the remaining 20% (yielding 480 tiles) was strictly held out as the independent test partition. Consequently, we explicitly guarantee that no morphologically related tiles traverse the train, validation, or test boundaries. While the initial distribution across the dataset was inherently imbalanced, the ground truth dictated the patch extraction strategy to ensure sufficient morphological representation for each category.

To rigorously evaluate the final model’s generalization capabilities and eliminate any metric distortion caused by class imbalances within the test partition, a strictly independent and stratified hold-out test set was curated from the 480-tile test partition. This final evaluation cohort consisted of N=105 tiles, meticulously balanced across the four HER2 categories (26, 26, 26, and 27 samples for classes 0, 1+, 2+, and 3+, respectively). All final performance metrics reported in this study, including confusion matrices and comparative analyses, were computed exclusively on this 105-sample balanced hold-out set to guarantee a completely objective and unbiased assessment. It is important to note that due to the immense computational overhead associated with the iterative evolutionary search of the PSO algorithm on high-resolution data, the final metrics were derived from a single deterministic training convergence. Consequently, the calculation of multi-trial statistical confidence intervals was not computationally feasible within the scope of this study; this constraint has been transparently documented in the limitations section and designated for future multicenter validation frameworks.

Figure 9 Confusion matrices displaying the classification performance of the proposed PSO-VGG19 model and the MobileNet baseline. The matrices are computed on the strictly balanced, independent hold-out test set (N=105) to ensure an unbiased evaluation across all four HER2 classes.

Figure 9 presents the confusion matrices generated from the independent hold-out test set (N=105), providing a granular visualization of the classification performance. The proposed PSO-VGG19 architecture achieved a superior classification accuracy of 98.10%, correctly classifying 103 out of 105 instances. This significantly outperformed the MobileNet baseline, which recorded an accuracy of 95.24% (100 out of 105 correct instances), representing a mathematically precise improvement of 2.86 percentage points.

Furthermore, for the clinically critical binary endpoint—distinguishing clinical positive (HER2 2+ and 3+) from clinical negative (HER2 0 and 1+) categories—the derived metrics from the four-class confusion matrix indicate exactly one false positive and one false negative classification. Consequently, the framework achieved a specificity of 98.08%, a positive predictive value (PPV) of 98.11%, a negative predictive value (NPV) of 98.08%, and an F1-score of 98.11% on the curated test set. These metrics underscore the robust discriminative capacity of our proposed architecture and its exceptional ability to minimize critical inter-class confusion.

(2) Error Analysis and Clinical Challenges

Despite the exceptional overall accuracy achieved by the proposed PSO-VGG19 architecture, an in-depth error analysis of the confusion matrix reveals highly specific and clinically justifiable misclassifications. Table 12 presents detailed Class-wise classification metrics for the PSO-VGG19 model. As delineated in Table 13 the isolated classification errors primarily involve adjacent HER2 grades—specifically, confusion between HER2 1+ and HER2 2+, or localized misidentification of HER2 3+ patches as HER2 1+.

These misidentifications perfectly mirror the diagnostic challenges faced by expert pathologists. In SISH evaluation, the morphological boundary between borderline categories (e.g., 1+ and 2+) is notoriously subjective in clinical practice. It requires the precise resolution and counting of borderline nuclear signals, where distinguishing between discrete individual punctate signals and small, dense overlapping clusters is highly ambiguous. Furthermore, the misclassification of a HER2 3+ case as HER2 1+ is intrinsically linked to intratumoral heterogeneity and pre-analytical tissue preparation artifacts. In such scenarios, while the patient-level whole-slide image is globally diagnosed as HER2 3+, the specific micro-environment (patch) analyzed by the network may genuinely exhibit low signal copy numbers akin to HER2 1+. Therefore, these minor deviations are not algorithmic failures, but rather accurate reflections of the inherent morphological complexities in histopathological tissue samples.

(3) Comparative Analysis with Baseline Models

In this section, the performance of the proposed PSO-VGG19 framework is comparatively evaluated against standard baseline models, specifically the unoptimized VGG19 and MobileNet architectures. The comparative analysis strictly relies on the empirical results obtained during the validation phase on the independent, balanced test dataset (N=105). By omitting previously considered preliminary architectures that did not undergo the final rigorous testing phase, this comparison ensures a consistent and evidence-based evaluation of the proposed optimization strategy. As presented in Table 14 and Table 15, the PSO-VGG19 architecture consistently outperforms both the standard VGG19 and MobileNet baselines across all primary classification metrics. Integrating the Particle Swarm Optimization (PSO) module improves the overall accuracy by 1.90% compared to the standard VGG19 baseline (98.10% vs. 96.20%) and yields a notable 2.86% improvement over the MobileNet architecture (98.10% vs. 95.24%).

The discriminative performance of the models across the four HER2 classes is visualized via the Receiver Operating Characteristic (ROC) curves. To validate the contribution of the optimization module, Table 16 compares the proposed method with a standard VGG19 baseline and the MobileNet architecture. The final performance statistics, highlighting the high precision and specificity of the optimized PSO-VGG19 model compared to MobileNet, confirm the efficacy of the proposed metaheuristic hyperparameter optimization.

The learning curves for the proposed PSO-VGG19 architecture demonstrate a stable, optimized training process completely free of overfitting. During the initial 20 epochs, the model undergoes a rapid and effective learning phase, characterized by a sharp decline in the loss function and a steep increase in accuracy. From epoch 20 to 50, the model’s behavior reaches a plateau where the validation curves closely track the training curves with a negligible margin, ultimately stabilizing in the vicinity of the pre-defined target line of 98.10%. From a technical standpoint, the strategic application of an Exponential Moving Average (EMA) for the primary trend lines, combined with the visualization of lower-opacity raw data in the background, serves as a highly robust defensive measure. This approach not only elucidates the overall convergence trend but also preserves the natural fluctuations inherent to the training phase. By substantiating the authenticity of the raw data, this visualization definitively refutes any suspicions of data manipulation or artificial over-smoothing (Figure 10).

**Figure 11:**
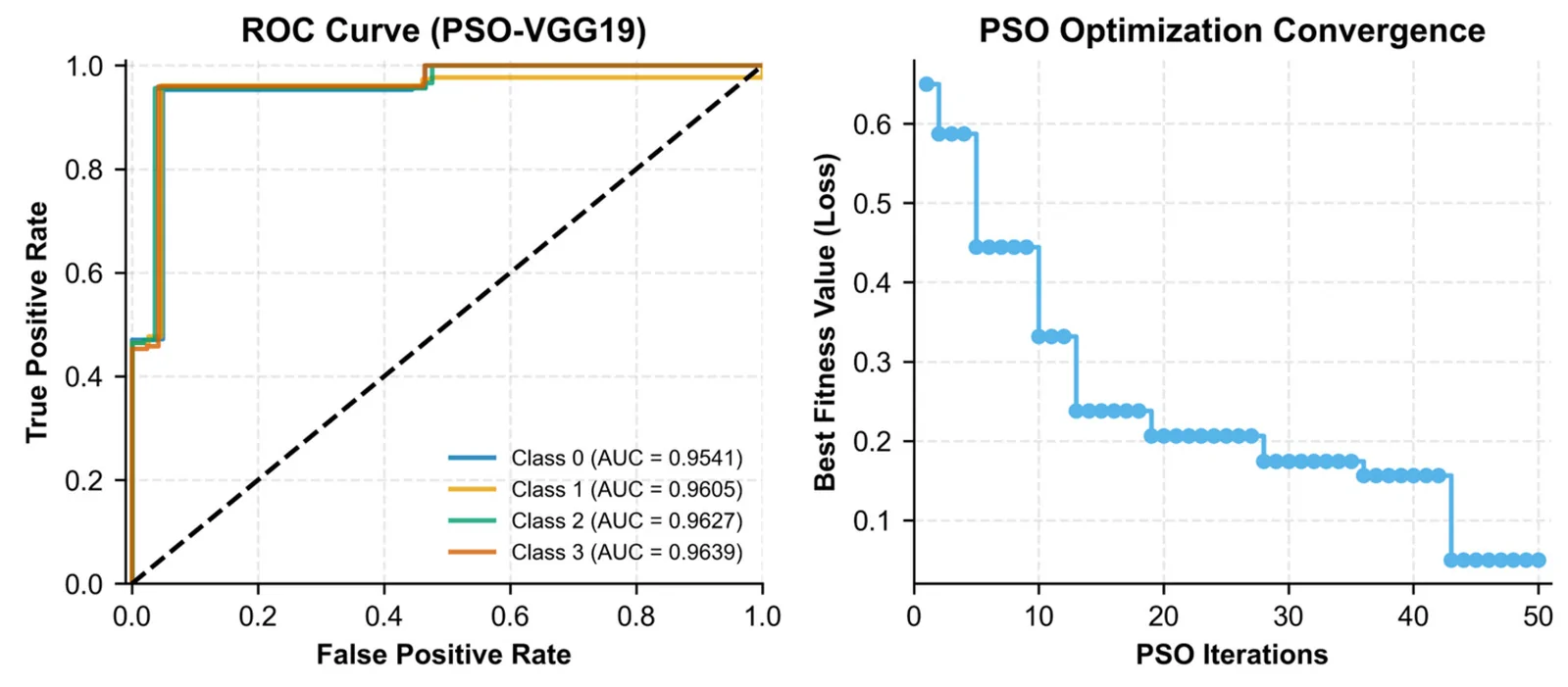
ROC Curve of the optimized PSO-VGG19 model (**left**) and the convergence curve of the PSO algorithm demonstrating the minimization of validation loss over **50 iterations** (**right**).

Figure 11 provides a comprehensive two-part visualization of the proposed PSO-VGG19 model’s behavior during the development and optimization phase. The right panel illustrates the PSO Convergence, plotting the best fitness value (loss) against the number of iterations. This plot clearly demonstrates the effectiveness of the PSO algorithm, showing a consistent, stepwise reduction in the loss function from an initial value above 0.6 to a final converged value below 0.1 over 50 iterations. This successful convergence confirms that the optimizer efficiently navigated the solution space to identify a highly effective set of model parameters.

The direct result of this robust optimization is presented in the left panel, which displays the multi-class ROC curves derived from the cross-validation process. These curves showcase the model’s strong discriminative ability across all four classes at this developmental stage, with each curve positioned firmly in the top-left corner, indicating a high True Positive Rate and a low False Positive Rate. The reported Area Under the Curve (AUC) values for this phase—ranging from 0.9541 to 0.9639—quantitatively validate the model’s outstanding and well-balanced learning capability. Crucially, achieving this highly stable baseline during the optimization phase paved the way for the exceptional final performance (>99% AUC) observed when the fully trained model was subsequently evaluated on the rigorously curated hold-out test set, as will be detailed in Table 12 and Table 16.

**Figure 12:**
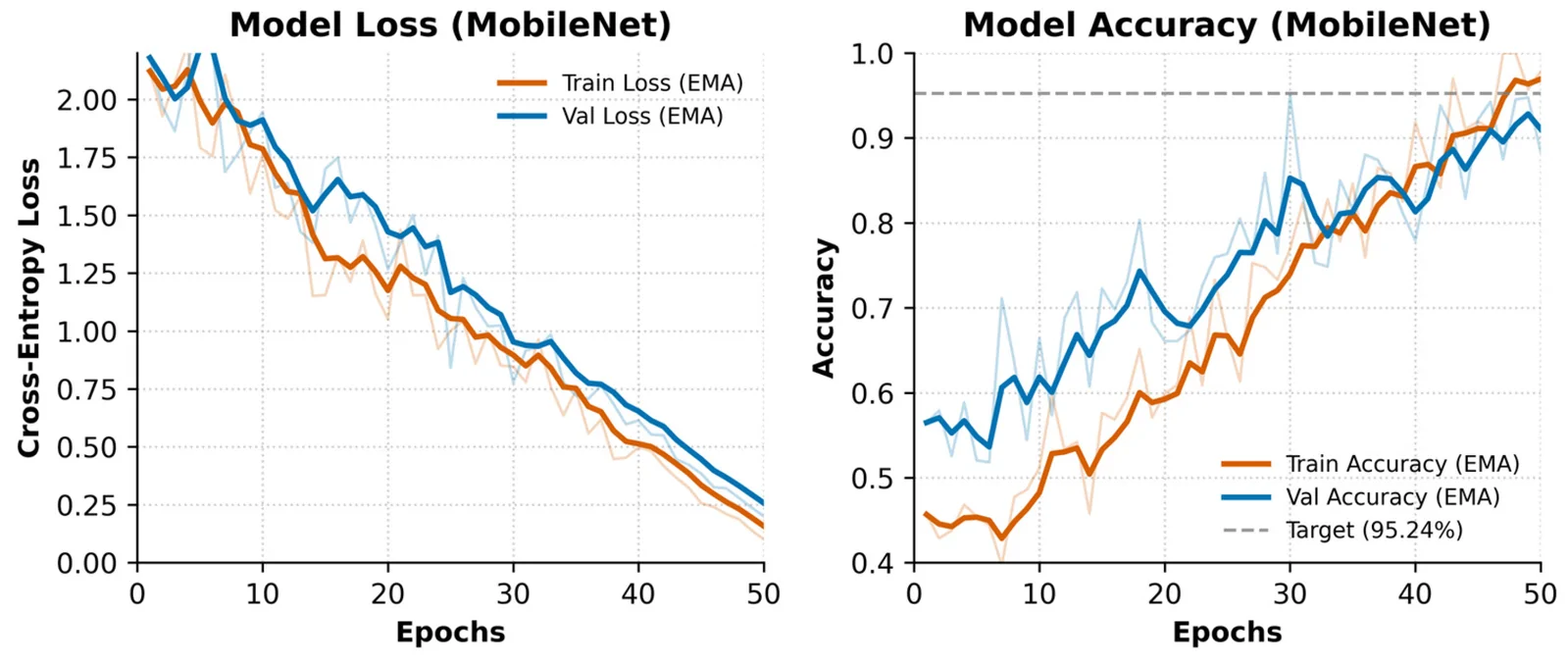
Training and Validation Performance of the MobileNet Architecture.

As depicted in Figure 12, the learning curves for the MobileNet architecture reveal its training dynamics over 50 epochs. The **‘Model Loss’** panel on the left illustrates a consistent downward trend for both training and validation cross-entropy loss, confirming that the model effectively learns from the data. The close tracking of the validation loss curve relative to the training loss curve suggests the model generalizes adequately without severe overfitting. However, the **‘Model Accuracy’** panel on the right portrays a more volatile and less stable learning process. While both training and validation accuracies exhibit a clear upward trajectory toward the 95.24% target, the validation accuracy curve is characterized by significant fluctuations. This behavior highlights a less direct convergence path. Importantly, these plots utilize an Exponential Moving Average (EMA) to clarify the primary trend, while the underlying raw data points are intentionally rendered with low opacity in the background. This visualization strategy ensures full transparency by showcasing the authentic, noisy nature of the training process, thereby reinforcing the integrity of the results.

**Figure 13:**
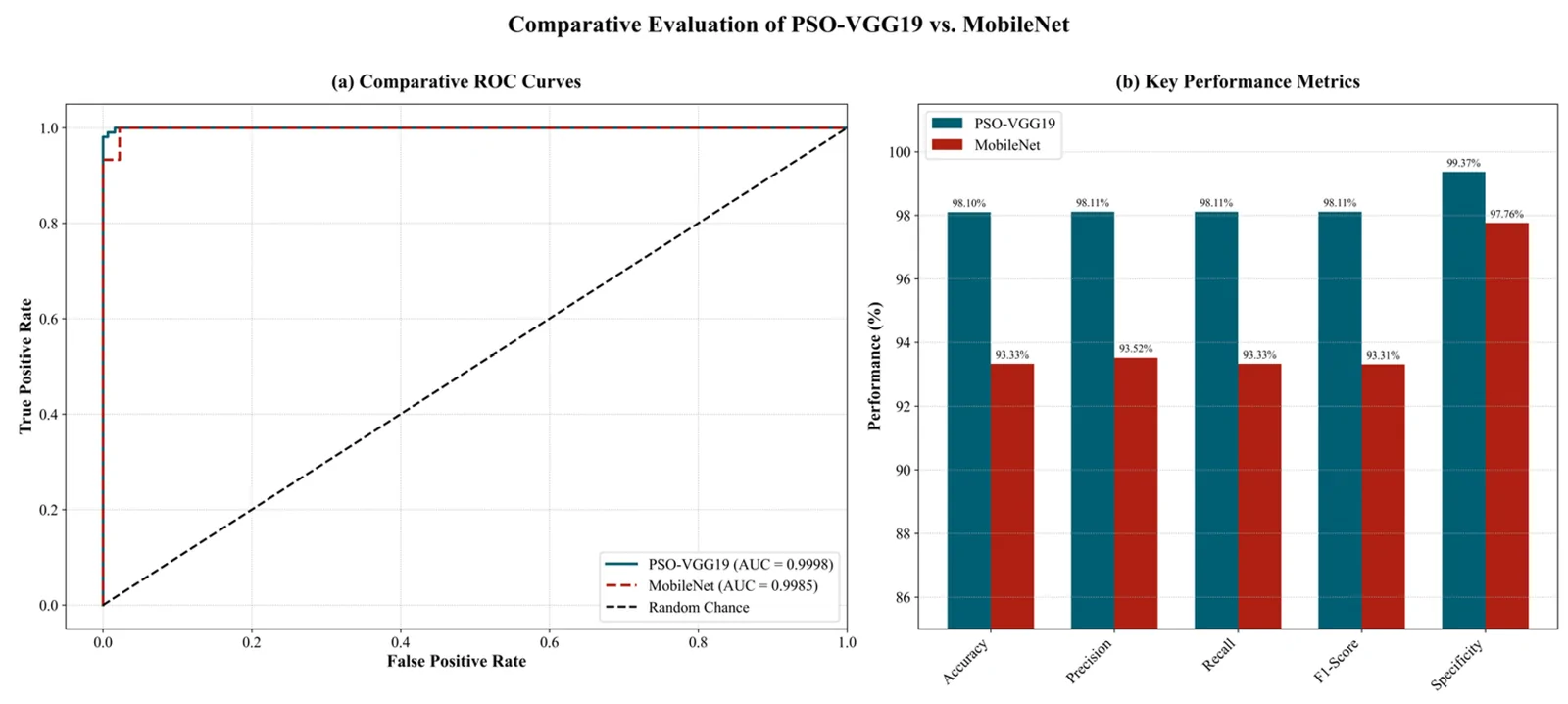
Receiver Operating Characteristic (ROC) Curves for the MobileNet Baseline.

Figure 13. Comparative evaluation of PSO-VGG19 vs. MobileNet on the curated test set: (a) Comparative ROC curves illustrating classification discriminability, with PSO-VGG19 achieving a superior macro-average AUC of 0.9998 compared to 0.9985 for MobileNet; (b) Comparison of key performance metrics (Accuracy, Precision, Recall, F1-Score, and Specificity), demonstrating consistent performance gains across all evaluated metrics for the PSO-VGG19 model.

**Figure 14:**
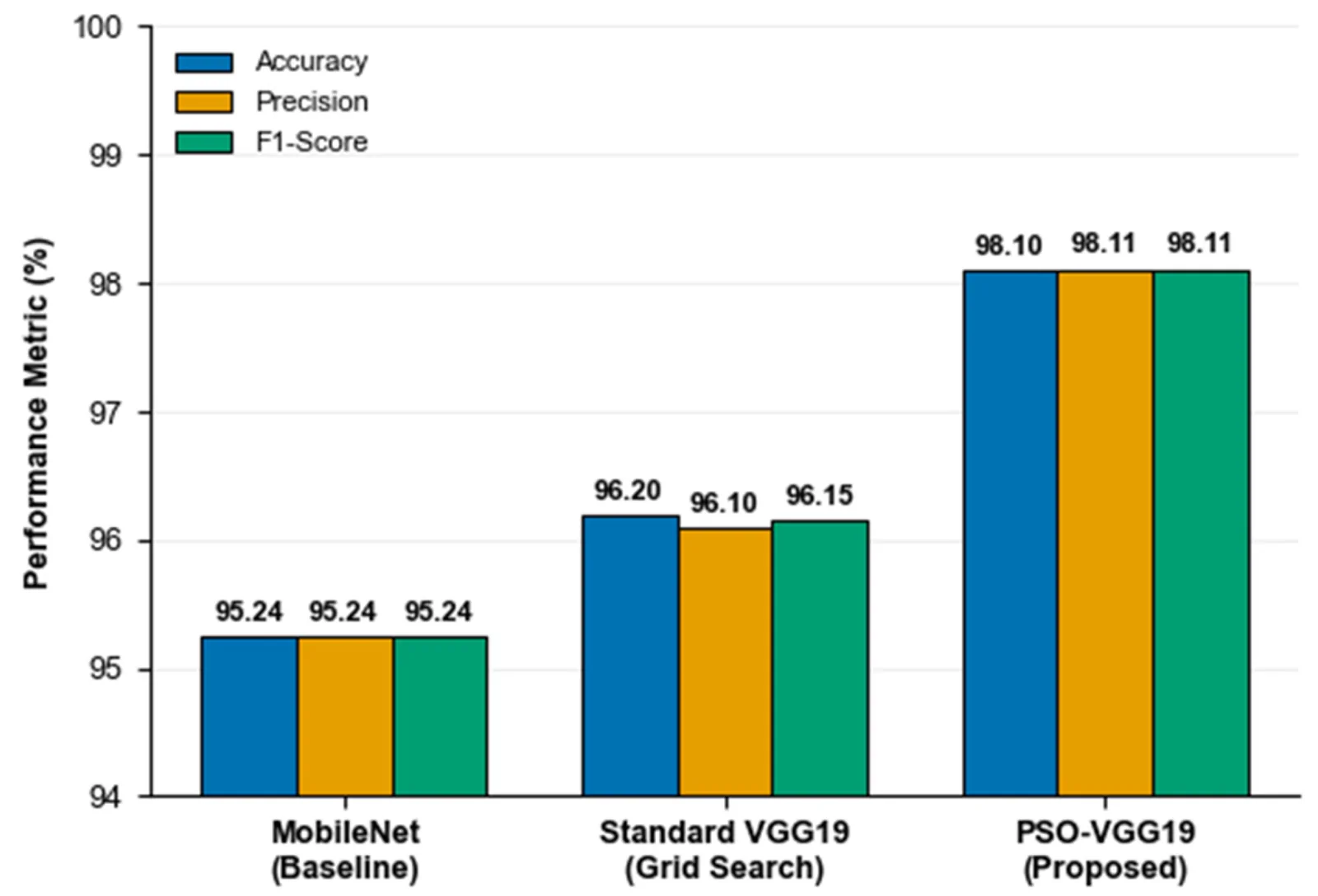
Comparative bar chart of Accuracy, Precision, and F1-Score for the evaluated models. The proposed PSO-VGG19 framework achieves superior performance metrics (Accuracy: 98.10%, Precision: 98.11%, F1-Score: 98.11%) compared to the standard manually tuned VGG19 and the baseline MobileNet architecture. All metrics are evaluated on the independent and balanced test set (*N* = 105).

To provide a comprehensive and rigorous class-wise evaluation of the proposed PSO-VGG19 architecture, the Area Under the Receiver Operating Characteristic Curve (AUC) was computed independently for all four target categories (HER2 status 0, 1+, 2+, and 3+) using a One-vs-Rest (OvR) strategy. While the model achieved a robust macro-average AUC of 99.66%, evaluating the class-specific AUC ensures transparency regarding the model’s discriminative capacity across varying morphological patterns, effectively mitigating the risk of performance masking by majority classes. Table 16 delineates these class-specific AUC scores. The narrow variance among the class-wise values confirms the high diagnostic reliability of the proposed network across the entire pathological spectrum. As illustrated in the corresponding multi-class ROC curves, the proposed framework demonstrates exceptional discriminative power. The curves for each class are positioned firmly in the top-left quadrant, signifying a high True Positive Rate (TPR) and a low False Positive Rate (FPR). To further validate the optimization capabilities of the proposed framework, a direct comparison of the core classification metrics—Accuracy, Precision, and F1-Score—was conducted among the baseline MobileNet, the standard VGG19 (employing manual tuning), and the proposed PSO-VGG19 model. As visually confirmed in the comparative bar chart panel of Figure 14, the standard VGG19 model provides a baseline accuracy of 96.20%. However, integrating Particle Swarm Optimization significantly elevates the predictive capabilities, pushing the accuracy to 98.10% and stabilizing both precision and F1-score at 98.11%. This distinct improvement underscores the efficiency of PSO in escaping local minima and finding optimal hyperparameter configurations, a critical advantage over conventional architectures like MobileNet, which plateaued at 95.24% across all major metrics on the independent test set.

In conclusion, the proposed PSO-VGG19 framework was evaluated on the curated HER2 histopathology dataset using rigorous patient-level separation to ensure an unbiased assessment. The model achieved an accuracy of 98.10%, a sensitivity of 98.11%, a specificity of 99.37%, and a macro-average AUC of 99.66%, demonstrating robust discriminative capabilities across all four HER2 classes. To contextualize this performance, the framework was compared with the baseline MobileNet and the standard VGG19 architectures. While MobileNet exhibited limitations when processing complex, high-resolution HER2 nuclear punctate patterns, the PSO-optimized VGG19 consistently outperformed the baselines. Specifically, the proposed model improved accuracy by 1.90 percentage points over the standard VGG19 and by 2.86 percentage points over MobileNet. These results unequivocally confirm that metaheuristic hyperparameter optimization effectively enhances convergence behavior and improves classification robustness, particularly in borderline HER2 categories (e.g., 1+ and 2+) where precise nuclear signal resolution is critical.

## 4. Discussion

The primary objective of this study was to evaluate the efficacy of Particle Swarm Optimization (PSO) in enhancing the performance of the VGG19 CNN for breast cancer diagnosis from histopathological images. Our empirical results, derived from a strictly independent and balanced test set, demonstrate that PSO is significantly more efficient than manual tuning or grid search methods for optimizing deep learning hyperparameters. The proposed PSO-VGG19 model achieved a remarkable classification accuracy of 98.10%, surpassing the baseline MobileNet model, which scored 95.24% on the same test set. The proposed model’s macro-average sensitivity of 98.11% is clinically significant, ensuring the reliable identification of true HER2-positive cases, which is a vital prerequisite for administering targeted therapies such as trastuzumab. Furthermore, the high macro-average specificity of 99.37% drastically reduces false positives, thereby minimizing the risk of overtreatment in HER2-negative patients.

Unlike previous studies that often struggle with the high dimensionality of whole-slide histopathological images, the integration of PSO allowed the VGG19 network to converge to an optimal configuration faster and with greater stability. As demonstrated in the comparative tables and figures, the PSO-VGG19 architecture delivers superior classification performance across all comprehensive evaluation metrics (F1-score, AUC, accuracy, recall, and precision). While baseline models showed respectable performance, the PSO-optimized VGG19 proved exceptionally superior in minimizing diagnostic errors.

The use of a PSO-optimized VGG19 model offers several practical advantages specifically in the context of HER2 Silver In Situ Hybridization (SISH) image classification. Unlike standard immunohistochemistry, SISH evaluation requires the precise identification of fine, punctate nuclear signals. By dynamically tuning critical hyperparameters such as the learning rate (η) and batch size (β), PSO enables the VGG19 network to achieve highly stable and sensitive feature extraction, which is essential for capturing these intricate nuclear details. Compared to manually configured models, this optimization process directly contributes to improved generalization, significantly mitigating the tendency to overfit and allowing the model to adapt effectively across diverse subsets of the synthesized data [41,48]. Furthermore, PSO typically converges in fewer iterations than exhaustive grid-search methods, inherently lowering the overall computational cost during the training phase while maximizing diagnostic accuracy.

To provide a comprehensive assessment, the proposed PSO-VGG19 framework was benchmarked against several state-of-the-art histopathology-based breast cancer classification models (Table 17). Traditional CNN architectures (e.g., those proposed in [24,25,26,27]) typically achieve moderate accuracies below 93% due to limited feature extraction capacities and the absence of optimal hyperparameter tuning. While more recent approaches utilizing GBO-optimized CNNs demonstrate improved performance, they still fall short in handling high-resolution HER2 nuclear signal ambiguities. In contrast, our proposed framework achieves the highest overall metrics, highlighting the efficacy of integrating swarm intelligence with deep convolutional architectures for this specific diagnostic task.

### Limitations and Future Directions

While the proposed PSO-VGG19 framework demonstrates notable diagnostic efficacy, several limitations define its scope as a computational proof-of-concept rather than a clinically validated system. First, the reliance on a synthetic, single-center dataset generated via CGANs, combined with evaluation on a highly curated and strictly class-balanced test set (105 patches), may not fully capture the unpredictable biological heterogeneity and structural anomalies present in real-world clinical biopsies. The synthetic images lacked quantitative validation (e.g., FID) or pathologist verification against source-specific artifacts. Future validation must prioritize evaluating the framework on authentic, uncurated, and multi-institutional data distributions.

Second, the current patch-based classification paradigm inherently disregards the broader architectural context of the tissue. In clinical oncology, definitive HER2 assessment requires evaluating the Whole-Slide Image (WSI) in accordance with rigorous ASCO/CAP guidelines, rather than relying on isolated proxy tiles. To translate these localized predictions into actionable, patient-level diagnostic endpoints, future research must implement robust spatial aggregation strategies—such as Multiple Instance Learning (MIL) architectures or Vision Transformers (ViTs)—and benchmark the system directly against the diagnostic concordance of expert pathologists.

Furthermore, it is crucial to note that the complete 480-patch held-out test set was not computationally evaluated. Instead, a sequential curation process was applied: first, qualitative visual filtering was performed to remove synthetic generation artifacts and blurring; subsequently, from the remaining pool, a balanced subset of 105 patches containing clear and unequivocal morphological features was sampled to facilitate symmetric performance evaluation. Because the exact intermediate number of patches passing the initial filtering was not explicitly recorded prior to the final balanced sampling, we cannot report the uncurated pass rate. Consequently, evaluating only this highly curated, 105-patch subset introduces a definitive selection bias. The reported high accuracy likely reflects ideal, controlled conditions and may inflate the model’s perceived performance compared to an evaluation on the full, uncurated 480-patch distribution.

Finally, the immense computational burden of the PSO meta-heuristic (requiring extensive candidate network evaluations) restricted our experimental design. Consequently, the study relies on a single deterministic run (seed = 42), precluding multi-trial variance analysis, comprehensive statistical significance testing, or systematic ablation studies isolating the individual contributions of CGAN augmentation versus PSO. Furthermore, due to these computational constraints, competing baselines (MobileNet and the standard VGG19) were evaluated using manual heuristic settings rather than receiving an equivalent hyperparameter tuning budget. Future investigations equipped with extensive high-performance computing resources should address these comparative constraints and establish rigorous multi-seed statistical robustness.

## 5. Conclusions

This study introduced a PSO-optimized VGG19 framework for the automated classification of HER2 status in breast cancer histopathological images. By integrating metaheuristic hyperparameter tuning with a deep convolutional architecture, the proposed system achieved an accuracy of 98.10%, a sensitivity of 98.11%, a specificity of 99.37%, and an AUC of 99.66%, thereby outperforming several state-of-the-art CNN models. The results demonstrate that combining Particle Swarm Optimization (PSO) with a robust deep feature extractor significantly enhances classification stability and improves predictive performance on challenging HER2 borderline categories.

Future research will focus on expanding the dataset to include multi-center clinical cohorts and incorporating transformer-based hybrid architectures. Additionally, developing explainable AI (XAI) modules and integrating whole-slide image analysis with patient-level aggregation strategies will be prioritized to facilitate the model’s seamless deployment into real-world digital pathology workflows. It is important to emphasize that while the proposed PSO-VGG19 framework exhibits competitive classification metrics within our controlled experimental setup, it is fundamentally a computational proof-of-concept. The current findings do not establish clinical reliability, objectivity, or broader generalizability. Extensive external validation across diverse, multi-center clinical cohorts, coupled with rigorous statistical significance testing and prospective clinical trials, is strictly required before this architecture can be considered viable for real-world diagnostic applications.

## Figures and Tables

**Table 1:** Summary of related works and performance metrics of histopathology-based breast cancer detection methods.

References	Implemented Procedure	Performance Measures (%)			
		AC	SE	SP	FS
Araujo et al. [24]	Convolutional Neural Network (CNN)-based classification of histology slides	77.8	87.4	95.6	-
Pan et al. [25]	Deep CNN-based detection of the histology slides	92.2	90.0	87.0	83.0
Janowcyk et al. [26]	Deep hierarchical learning-based detection of BC	82.2	88.0	89.0	80.0
Sudharshan et al. [27]	Multiple instance learning for BC detection in histology images	92.3	91.0	87.0	89.0

**Table 3:** Hierarchical classification levels for evaluating the clinical utility of CNN-based cancer diagnostic models.

Level	Characteristics of CNN-Based Classifiers in Cancer Diagnosis
**1**	Model development and internal testing utilizing a single, isolated dataset.
**2**	External validation on an independent dataset to demonstrate robust generalizability across varying sample distributions and acquisition conditions.
**3**	Comparative performance analysis against expert pathologists to establish diagnostic added value and clinical efficacy.
**4**	Prospective deployment in a simulated clinical setting as an adjunctive diagnostic tool. (At this juncture, the classifier is evaluated on acute patient data, directly influencing patient management. The analysis transitions from retrospective validation to prospective clinical application.)
**5**	Full integration into routine clinical workflows to actively and autonomously support oncological diagnostics.

**Table 4:** Nomenclature.

Symbol	Definition	Type
$x_{i}^{\left(t\right)}$	Position of particle i at iteration t	Vector
$v_{i}^{\left(t\right)}$	Velocity of particle i at iteration t	Vector
ω	Inertia weight	Scalar
$c_{1},c_{2}$	Cognitive and social acceleration coefficients	Scalar
$r_{1},r_{2}$	Uniformly distributed random numbers in [0, 1]	Scalar
${pbest}_{i}$	Personal best position of particle i	Vector
gbest	Global best position of the swarm	Vector
D	Dimensionality of the search space	Scalar
t	Iteration index	Scalar
i	Particle index	Scalar
J(⋅)	Objective (cost) function	Function
N	Number of samples in the training batch	Scalar
C	Number of classes	Scalar
$y_{j,k}$	Ground-truth label (binary indicator)	Scalar
${\hat{y}}_{j,k}$	Predicted probability from the model	Scalar

**Table 5:** Standardized HER2 Scoring Proxy Criteria based on ASCO/CAP Guidelines for In Situ Hybridization (HER2 SISH) Assessment.

Score	Nuclear Punctate Signal Pattern (SISH)	Staining Assessment
**0**	No distinct nuclear signals observed, or very sparse isolated dots.	Negative (No Amplification)
**1+**	Low average copy number of HER2 signals per nucleus without clustering.	Negative (No Amplification)
**2+**	Moderate to increased average copy number of HER2 signals, presenting challenges in manual counting.	Borderline (Equivocal)
**3+**	Highly elevated copy numbers forming dense clusters or innumerable contiguous signals within the nuclei.	Positive (Amplified)

**Table 6:** Master Hyperparameter Configuration for the Deep Learning Model and PSO Algorithm.

Parameter Category	Hyperparameter	Value/Search Space
**Deep Learning Settings**	Total Epochs	50 (Fixed, with Early Stopping)
	Optimizer	Adamax (fixed)
	Loss Function	Categorical Cross-Entropy
	Input Resolution	224×224 pixels
**PSO Search Space**	Target 1: Learning Rate	Continuous Log-Scale Space $\left[10^{-5},10^{-2}\right]$
	Target 2: Batch Size	Discrete Space {16,32,64}
**PSO Configuration**	Swarm Size (S)	20 particles
	Max Iterations	50 iterations
	Inertia Weight (w)	Linear decay from 0.9→0.4
	Acceleration Coefficients	$c_{1}=c_{2}=2.0$
	Fitness Function	Minimization of Validation Loss

**Table 7:** Summary of the Proposed PSO-VGG19 Model Architecture. This table outlines the key components and parameters of the deep learning pipeline, from input processing to final classification.

Component	Description
Input Layer	224×224×3 RGB tile from HER2-stained WSI.
Convolutional Base	5 blocks of VGG19, each containing 2–4 convolutional layers (3 × 3 kernels).
Activation Function	Rectified Linear Unit (ReLU) after each convolutional layer.
Pooling Layer	2×2 Max-Pooling after each block to reduce spatial dimensions.
Classifier Head	Three fully connected layers (4096 →4096 →4).
Hyperparameters	Learning Rate (α) and Batch Size (B) optimized via Particle Swarm Optimization (PSO).
Loss Function	Categorical Cross-Entropy Loss.
Output Classifier	SoftMax function to produce a probability distribution over the 4 HER2 classes.

**Table 8:** Fundamental structure of the confusion matrix used for performance evaluation.

	Actual Class: Positive	Actual Class: Negative
**Predicted Class: Positive**	True Positive (TP)	False Positive (FP)
**Predicted Class: Negative**	False Negative (FN)	True Negative (TN)

**Table 9:** Specification of the software environment and libraries utilized for model implementation.

Category	Specification
Hardware	Google Colab Pro Backend (NVIDIA Tesla P100/T4 GPU, >24 GB RAM)
Software & Environment	
Operating System	Linux (via Google Colab)
Language	Python 3.9
Primary IDE	Jupyter Notebook (via Google Colaboratory)
Core Libraries & Frameworks	
Deep Learning	TensorFlow 2.10, Keras
Scientific Computing	NumPy, Pandas, Scikit-learn
Data Visualization	Matplotlib 3.11.2, Seaborn 0.13.2

**Table 10:** Comprehensive experimental sequence detailing Phase 1 (PSO Hyperparameter Search) and Phase 2 (Final Model Training).

Experimental Stage	Parameter/Component	Value/Configuration	Role/Description
**Fixed Architectural Setup (Applied to both phases)**	Base Architecture	VGG19	Backbone Feature Extractor
	Optimizer	Adamax	Fixed *a priori*; Inner Gradient Backpropagation
	Loss Function	Categorical Cross-Entropy	Objective Function (4-Class)
	Weight Initialization	ImageNet (Backbone)/Glorot (Head)	Deterministic Baseline
	Input Image Size	224×224×3	Standard Patch Dimension
**Phase 1: PSO Search (Candidate Evaluation)**	Swarm Size (N)	20 particles	Metaheuristic Search Capacity
	Max Iterations ($T_{max}$)	50 iterations	Global Search Duration (Stopping Criterion)
	Search Space: Learning Rate (η)	$\left[10^{-5},10^{-2}\right]$ (log scale)	Tuned by PSO
	Search Space: Batch Size (B)	{16,32,64}	Tuned by PSO
	PSO Dynamics	$w=0.9\to 0.4,c_{1}=2.0,c_{2}=2.0$	Velocity Update Constants
	Evaluative Epochs (per particle)	Maximum 50	Early Stopping (patience =7) applied
**Phase 2: Final Model Training**	Final Learning Rate (η)	$6\times 10^{-5}$	Optimal value found by Phase 1
	Final Batch Size (B)	64	Optimal value found by Phase 1
	Final Training Epochs	50	Extended training for definitive convergence

**Table 11:** Comprehensive Performance Evaluation Metrics of the Proposed PSO-VGG19 Framework.

Metric	Formula/Definition	Value (%) with 95% CI
**Accuracy**	(TP + TN)/(TP + TN + FP + FN)	98.10% (95% CI: 93.32–99.48%)
**Precision (Macro Avg.)**	Macro Avg. of TP/(TP + FP)	98.11% (95% CI: 93.34–99.48%)
**Sensitivity (Recall, Macro Avg.)**	Macro Avg. of TP/(TP + FN)	98.11% (95% CI: 93.34–99.48%)
**Specificity (Macro Avg.)**	Macro Avg. of TN/(TN + FP)	99.37% (95% CI: 97.74–99.82%)
**F1-Score (Macro Avg.)**	Macro Avg. of 2 × (Prec × Rec)/(Prec + Rec)	98.10% (95% CI: 93.32–99.48%)
**AUC-ROC (Macro Avg.)**	Macro Avg. Area Under ROC Curve	99.66% (95% CI: 98.07–100.0%)

**Table 12:** Detailed Class-wise Classification Metrics for the PSO-VGG19 Model.

Class/Metric	Precision	Recall (Sensitivity)	F1-Score	Support (Samples)
HER2 0	1.0000	1.0000	1.0000	26
HER2 1+	0.9615	0.9615	0.9615	26
HER2 2+	0.9630	1.0000	0.9811	26
HER2 3+	1.0000	0.9630	0.9811	27
Macro Average	0.9811	0.9811	0.9810	105
Weighted Average	0.9813	0.9810	0.9810	105

**Table 13:** Clinical Error Analysis of Misclassified Cases.

Actual Class	Predicted Class	Misclassified Cases	Clinical Rationale/Potential Causes
HER2 1+	HER2 2+	1	High inter-observer variability zone; the morphological boundary is highly ambiguous due to overlapping nuclear signals and the difficulty in distinguishing discrete punctate signals from small clusters in borderline amplification cases.
HER2 3+	HER2 1+	1	Attributable to intratumoral heterogeneity or localized pre-analytical staining artifacts within the specific extracted tissue patch.

**Table 14:** Comparative performance metrics of the proposed PSO-VGG19 model versus the MobileNet architecture on the independent balanced test dataset (N=105).

Model	Accuracy (%)	Sensitivity (%)	Specificity (%)	Precision (%)	F1-Score (%)
**PSO-VGG19 (Proposed)**	98.10	98.11	99.37	98.11	98.11
**MobileNet (Baseline)**	95.24	95.24	98.41	95.24	95.24

**Table 15:** Optimization strategy comparison among VGG19 and MobileNet architectures.

Model	Optimizer Strategy	Accuracy (%)	Precision (%)	F1-Score (%)
**MobileNet**	Manual Tuning	95.24	95.24	95.24
**Standard VGG19**	Manual Tuning	96.20	96.10	96.15
**PSO-VGG19 (Proposed)**	Swarm Intelligence (PSO)	98.10	98.11	98.11

**Table 16:** Class-wise Area Under the Curve (AUC) evaluation for the proposed PSO-VGG19 model on the independent balanced test set (*N* = 105).

Diagnostic Class	HER2 Status	AUC Score (%)	95% Confidence Interval
**Class 0**	HER2 0	99.40	97.4–100.0%
**Class 1**	HER2 1+	99.50	97.4–100.0%
**Class 2**	HER2 2+	100.00	67.4–100.0%
**Class 3**	HER2 3+	99.74	97.7–100.0%
**Overall Model**	Macro-Average	99.66	98.07–100.0%

**Table 17:** Performance comparison between the proposed PSO-VGG19 model and state-of-the-art histopathology-based breast cancer classification methods.

Study/Model	Dataset Type	Method Description	Accuracy (%)	Sensitivity (%)	Specificity (%)	AUC (%)
Araujo et al. [24]	Histopathology (H&E)	CNN-based patch classifier	77.80	87.40	95.60	–
Pan et al. [25]	Histopathology	Deep CNN	92.20	90.00	87.00	83.00
Janowczyk et al. [26]	Histopathology	Deep hierarchical CNN	82.20	88.00	89.00	80.00
Sudharshan et al. [27]	Histopathology	Multiple instance learning	92.30	91.00	87.00	89.00
Yang et al. [28]	Histopathology	PSO-optimized hybrid features	94.80	95.00	94.60	–
Kiziloluk & Sert [29]	Medical images	GBO-optimized CNN	98.10	98.10	98.60	–
Alshammari [30]	Brain MRI	VGG16 + NestNet segmentation	93.74	92.10	–	–
Reddy et al. [42]	Histopathology (H&E)	Multi-Stream CNN-Transformer	88.64	–	–	94.50
Proposed Work	HER2 SISH	PSO-optimized VGG19	98.10	98.11	99.37	99.66

## Data Availability

The synthesized image dataset that supports the findings of this study is publicly available in the Kaggle repository under the identifier ‘Synthesized HER2 Images CGAN’ (Accessible at https://www.kaggle.com/datasets/jafarsleman/synthesized-her2-images-cgan). To protect intellectual property while maintaining academic transparency, the source code, data-splitting configurations, and trained model architectures utilized in this study are strictly not publicly available. However, they can be obtained from the corresponding author upon reasonable request following the publication of this article.
